# A knowledge-based *T*^2^-statistic to perform pathway analysis for quantitative proteomic data

**DOI:** 10.1371/journal.pcbi.1005601

**Published:** 2017-06-16

**Authors:** En-Yu Lai, Yi-Hau Chen, Kun-Pin Wu

**Affiliations:** 1 Institute of Biomedical Informatics, National Yang-Ming University, Taipei 11221, Taiwan; 2 Bioinformatics Program, Taiwan International Graduate Program, Institute of Information Science, Academia Sinica, Taipei 11529, Taiwan; 3 Institute of Statistical Science, Academia Sinica, Taipei 11529, Taiwan; University of Richmond, UNITED STATES

## Abstract

Approaches to identify significant pathways from high-throughput quantitative data have been developed in recent years. Still, the analysis of proteomic data stays difficult because of limited sample size. This limitation also leads to the practice of using a competitive null as common approach; which fundamentally implies genes or proteins as independent units. The independent assumption ignores the associations among biomolecules with similar functions or cellular localization, as well as the interactions among them manifested as changes in expression ratios. Consequently, these methods often underestimate the associations among biomolecules and cause false positives in practice. Some studies incorporate the sample covariance matrix into the calculation to address this issue. However, sample covariance may not be a precise estimation if the sample size is very limited, which is usually the case for the data produced by mass spectrometry. In this study, we introduce a multivariate test under a self-contained null to perform pathway analysis for quantitative proteomic data. The covariance matrix used in the test statistic is constructed by the confidence scores retrieved from the STRING database or the HitPredict database. We also design an integrating procedure to retain pathways of sufficient evidence as a pathway group. The performance of the proposed *T*^2^-statistic is demonstrated using five published experimental datasets: the T-cell activation, the cAMP/PKA signaling, the myoblast differentiation, and the effect of dasatinib on the BCR-ABL pathway are proteomic datasets produced by mass spectrometry; and the protective effect of myocilin via the MAPK signaling pathway is a gene expression dataset of limited sample size. Compared with other popular statistics, the proposed *T*^2^-statistic yields more accurate descriptions in agreement with the discussion of the original publication. We implemented the *T*^2^-statistic into an R package T2GA, which is available at https://github.com/roqe/T2GA.

## Author summary

Pathway analysis is a common approach to quickly access the pathways being regulated in the experiments. There are numerous statistics to perform pathway analysis; most of them assume that the genes or proteins are independent of each other for statistical ease. This assumption, however, is unrealistic to the real biological system and may cause false positives in practice. A standard way to address this issue is to measure the associations among genes or proteins. Unfortunately, the estimation of associations requires sufficient sample size, which is usually not available for proteomic data produced by mass spectrometry. In this study, we propose a *T*^2^-statistic, which estimates the associations among gene products, to perform pathway analysis for quantitative proteomic data. Instead of calculating the associations directly from data, we use the confidence scores retrieved from protein-protein interaction databases. We also design an integrating procedure to reserve pathways of sufficient evidence as a regulated pathway group. We compare the proposed *T*^2^-statistic to other popular statistics using five published experimental datasets, and the *T*^2^-statistic yields more accurate descriptions in agreement with the discussion of the original papers.

## Introduction

Great progress has been made toward the development of high-throughput technologies and their application to biological and clinical research. In a quantitative experiment, genes or proteins with significant changes in expression are potential to have important roles in a given phenotype or phenomenon. Therefore, the analysis of quantitative experimental data generally produces a list of differentially expressed genes or proteins in order. The list may share some insights if the aim of the experiments is restricted to few targets. As regards high-throughput data, the list hardly provides biological understanding of the mechanisms being studied, since the data involve complicated regulations among biomolecules and the number of biomolecules is too large to examine all candidates individually. Systematically investigating the underlying mechanisms from a high-throughput data therefore become a new challenge.

To confront the challenge, one idea is to apply *pathway analysis* to identify the genes or proteins that are known to be involved in a biological process or interaction based on the existing knowledge. Many approaches of pathway analysis have been developed over years concerning different methodologies. Some general reviews of pathway analysis approaches can be found in [1–4]. These approaches can be broadly divided into two major factions: a competitive null with a gene sampling model and a self-contained null with a subject sampling model. The null hypothesis of *a competitive test* suggests that the target pathway (or a predefined gene set) is differentially expressed as well as the rest of all pathways. Practically a competitive null is closely tied to (although not necessarily) a gene sampling approach [5]. *A gene sampling model* principally implies *the independence assumption*; the assumption presupposes that genes are expressed independently of each other so that these genes can be manipulated as the sampling subject to produce the null distribution. The null hypothesis of *a self-contained test*, on the other hand, suggests that the target pathway is not differentially expressed between distinct phenotypes. Using the phenotypes of the experiments as the sampling subject is the setup of *a subject sampling model*. In other words, a competitive null with a gene sampling approach let pathways compete with each other in order to rank these pathways and use the number of genes as the sample size in the meanwhile; whereas a self-contained null with a subject sampling approach examine each single pathway to determine if the pathway is indeed differentially expressed between phenotypes and use the number of experiments as the sample size. Intuitively, a competitive null aims to find the pathway that is most significant among all pathways; a self-contained null aims to find the pathway that is the most significantly expressed between phenotypes. The classification of null hypotheses and sampling methods is firstly suggested by Geoman et al [5]. Even both categories have their own pitfalls and benefits, the authors suggested using a self-contained null with a subject sampling model in pathway analysis. Competitive null with gene sampling model usually implies the independence assumption which may produce inaccurate small *p*-values, cause serious false positives [6], and result misleading interpretations [5]. Further methodology issues of using a self-contained null can be found in [7–9], and of using a competitive null can be found in [10].

Another issue of pathway analysis comes from the construction of test statistics. These statistics can be divided into *univariate tests* and *multivariate tests*. Univariate statistics, such as a modification or a weighted summation of the *t*-scores [11–14], only focus on expression of genes or proteins and assume these biomolecules as independent units for statistical ease. The independence assumption ignores the associations among biomolecules with similar functions or cellular localization, as well as the interactions among them manifested as changes in expression ratios. In contrast, multivariate statistics take into consideration the associations among genes or proteins [15–20]. Some methodology studies [9, 21–23] have evaluated univariate tests and multivariate tests with synthetic and experimental datasets. Compared with multivariate tests, univariate tests generally result a decrease in statistical power [21] and an increase in false positive rate [22] along with the rise of average correlation. Multivariate approaches usually incorporate the sample covariance matrix into calculation to address biological interaction. However, the sample covariance may not be precise enough to estimate the associations if the sample size is very limited. Compared with other gene expression data, proteomic data produced by mass spectrometry are more difficult to analyze systematically due to the limited number of experiments. This limitation causes current multivariate tests incompetent because the sample covariance will not be a robust statistic.

The composition of pathway diagrams also become a challenge to pathway analysis. A *pathway* is a group of biomolecules that participate in a particular cellular process. The members of a pathway are usually defined by the tradition (i.e., the history of pathway discovery) of molecular biology scientists. The structure of pathway diagrams is not standardized and therefore arises some issues to pathway analysis. First, the same pathway from different databases or other sources may have the same core members but different side members. Under different experimental conditions, the size of accessible biomolecules also changes. For example, the phosphorylation proteomic data may not provide information to the proteins not belong to phosphoproteome. Since the number of members within a pathway is not a constant, using this number as a parameter to determine if the pathway is significant or not may lead to inconsistent results. This issue arises with the assumption of a competitive null. Second, some molecules appear over pathways may play important roles as communication centers. For example, *p53* appears in 38 pathways in the KEGG database. The shared members may interact or cooperate with each other and form a functional module. If this module is regulated (i.e., the members within the module are differentially expressed as they cooperate together), the subsets of this module may present in abundant pathways and make these pathways seemingly significant. To identify the regulated pathway among the significant pathways of common modules, biologists usually utilize the distinctive molecules participating in that specific pathway; whereas pathways do not contain distinctive molecules may be irrelevant to the underlying mechanism. However, most of the current approaches do not take consideration of this issue. The suggestion of irrelevant pathways due to the redundancy over the significant pathways usually causes confusion to data description. A recent study [24] also focuses on the second issue. They demonstrate how the shared members affect *p*-values, and try to address this problem under a competitive null.

In this study, we introduced a multivariate test, based on the Hotelling’s *T*^2^-statistic, to perform pathway analysis for quantitative proteomic data. The most serious problem of analyzing proteomic data produced by mass spectrometry is the limited number of experiments. We usually obtain only a few replicates (biological or technical) per experimental condition. To manage this issue, we had two special designs in our test. First, instead of using the sample covariance matrix (which is not robust when the sample size is limited), we use the covariance matrix that is constructed of the probabilistic confidence scores provided by the STRING and HitPredict databases. The proposed *T*^2^-statistic is then built of the protein expression profile and the covariance matrix to consider the expression level of individual proteins along with the associations among them. Second, we designed a self-contained model to produce a null distribution of altering protein expression while retaining the structure of protein associations. We are not capable of applying a subject sampling model because the number of experiments is too limited. In addition to the settlement of sample size issue, we designed an integrating procedure to categorize significant pathways as well as to avoid redundancy. The performance of the proposed *T*^2^-statistic is demonstrated using five public experimental datasets with different levels of biological complexity: the T-cell activation, the cAMP/PKA signaling, the effect of dasatinib on the BCR-ABL pathway, the differentiation process of myoblast, and the protective effect of myocilin via the MAPK signaling pathway. The first four datasets are proteomic data produced by mass spectrometry; the last dataset is a gene expression data of low sample size. We compared *T*^2^ with other popular statistics: DPA [25], GSEA [26], DAVID [27, 28], and IPA [29]. For most of the situations, *T*^2^ yielded more accurate descriptions in agreement with the discussion of the original publication.

## Materials and methods

### Experimental data

We took four proteomic datasets of different biological complexity and experimental properties to demonstrate our approach. To be comprehensive, the testing datasets include the case of pathway activation and inhibition; also the case of signaling phosphoproteome and cellular proteome. We only used the final ratios provided by the datasets since they may have different integration approaches (e.g. to combine the results of biological and/or technical repeats, to handle missing data or outliers) under different experimental designs. The summarized ratio is also the most available format for quantitative proteomic data. In this situation, the sample size of data becomes only one, calculating a covariance matrix is not even possible. Our approach provide a solution to undertake this difficulty. We also applied our approach on a gene expression dataset of three samples to demonstrate that the general idea is applicable to other quantitative data of low sample size.

#### The phosphoproteomic data of TCR signaling

The T-cell receptor (TCR) signaling phosphoproteomic data [30] aim to reveal system-wide phenomena associated with T cell activation. The authors used OKT3, an antibody specific to CD3*ϵ*, to initiate the TCR signaling transduction in the human leukemia cell line Jurkat T lymphocyte. They used stable isotope labeling by amino acids in cell culture (SILAC) method to perform large-scale quantitative phosphoproteomic analyses and identified 696 TCR-responsive phosphorylation sites on 453 proteins. Phosphopeptides showing more than 1.85-fold change in abundance are qualified as “TCR-responsive” sites, suggested by the authors under their experimental conditions. We used these 696 proteins since the original publication does not provide raw data, and we aimed to use this small dataset as a positive control to evaluate the proposed statistic. The dataset contains three time points: 5, 15, and 60 min; the number of UniProt accessions are 30, 376, and 330, respectively. The authors specifically enriched pTyr-contained peptides in 5 and 15 min experiments since pTyr constitutes less than 1% of the total amino acid content; a global phosphopeptide-enrichment approach was used for 15 and 60 min experiments. The 5 min experiment is extremely small because it contained only pTyr-enriched peptides.

#### The phosphoproteomic data of cAMP/PKA signaling

The cAMP-dependent protein kinase (i.e. protein kinase A, PKA) signaling phosphoproteomic data [31] aim to provide a resource of substrates of PKA. The authors also used SILAC to profile quantitative changes of potential PKA substrates. Prostaglandin E2 (PGE2) was applied to increase intracellular cAMP and activate PKA in Jurkat T cells. The light cells (L) was used for control, the medium (M) and the heavy (H) were treated with PGE2 for 1 and 60 min, respectively. The authors identified 4284 phosphorylation sites on 607 proteins, and they discussed the dynamic phosphorylation upon stimulation by PGE2. The study considered two expression ratios: M/L and H/L; the number of UniProt accessions are 594 and 595, respectively.

#### The cellular proteomic data of myogenesis

This cellular proteomic data [32] monitor the changes in protein expression underlying the phenotypic conversion of human primary myoblasts; from primary mononucleated muscle cells to multinucleated myotubes, using the SILAC method. The authors used human satellite cells isolated from a quadriceps muscle biopsy of a 5-day old infant. The light (L), medium (M) and heavy (H) cells were first treated with the growth medium (serum-supplemented; low glucose), then switched to the differentiation medium (serum-free; high glucose) for 0, 24, and 72 hr, respectively. This study quantified 2240 proteins, in which 2227 unique UniProt identifiers are quantified for both M/L and H/L expression ratios.

#### The phosphoproteomic data of BCR-ABL signaling for CML treatment

The pathology of chronic myeloid leukemia (CML) is commonly associated with an oncogenic tyrosine kinase BCR-ABL. Dasatinib is an inhibitor of the BCR-ABL and Src family tyrosine kinase, and it serves as a clinical drug for treatment of CML. This phosphoproteomic data [33] aim to inspect the effects of dasatinib on the entire cell signaling network, using the SILAC method. The authors used the human leukemia cell line K562, which expresses the activated BCR-ABL fusion protein, to examine cellular phosphorylation levels for three conditions. For one hour, the light cells (L) was treated with DMSO only, the medium (M) and the heavy (H) were treated with 5 and 50 nM dasatinib, respectively. The authors identified 5063 phosphorylation sites on 1889 proteins, and they further discussed the mechanisms induced by dasatinib. The study considered two expression ratios: M/L and H/L; the number of UniProt accessions are 5453 and 5443, respectively.

#### The gene expression data of MAPK signaling

The MAPK signaling gene expression data [34] aims to understand the functions of myocilin, a causative gene for open angle glaucoma. The authors suggested that myocilin promotes cell proliferation and resistance to apoptosis via the ERK1/2 MAPK signaling pathway. The microarray analysis compared a stably transfected HEK293 cell line expressing myocilin with the control cell lines. The cells were treated with the 10*μ*m MEK inhibitor U0126 two hours prior to the induction of myocilin expression. The gene expression profiling was performed using Human Affymetrix Gene Chip U133 Plus version 2.0. The processed dataset includes three treatment and three control expression values. We took the mean of the triplicated data as the representative expression. Then we took the difference of the treatment group and the control group since the processed data is already normalized. We used the identifier mapping files from the official websites and the final data include 21816 unique UniProt identifiers.

### Databases

To provide more generalized results, we choose two pathway databases and two protein-protein interaction databases: *KEGG* and *Reactome* provide pathway categories served as predefined gene sets, *STRING* and *HitPredict* contributes the confidence scores to estimate the covariance between protein expressions.

#### KEGG

The *Kyoto Encyclopedia of Genes and Genomes* (KEGG) pathway database [35] collects manually drawn pathway maps in various topics, especially in metabolism. We downloaded the pathway database in the KGML format from the KEGG website. We used the version last updated on 5th July 2016. The human pathway database (the organism code: *hsa*) contains 303 pathway diagrams. We excluded the overview maps since they depict very general biological concepts (e.g. one overview map is titled “Metabolic pathways”). There remain 290 pathways for the following analysis. For each pathway, we retrieved the components tagged as *gene*, *enzyme*, and *group*. Those components are associated with gene products so we use them to map the identifiers of experimental data onto pathways.

#### Reactome

The *Reactome* pathway database [36] provides curated pathways of signal transduction, transport, DNA replication, protein synthesis, metabolism and other cellular processes. We downloaded the pathway database in the tab-delimited format from the Reactome website. We used the version last updated on 30th June 2016 (v57). The human pathway database (the organism code: *9606*) contains 1618 pathway diagrams of the lowest level (i.e. most specific) and we only used the lowest level pathways for the following analysis.

#### STRING

The STRING database [37] scores and integrates known and predicted protein-protein interactions and gives a global perspective for many organisms. STRING provides eight PPI types: *neighborhood, gene fusion, co-occurrence, co-expression, experiments, databases, textmining* and *homology* [38]. STRING suggests four levels of confidence score: *low confidence* (0.150), *medium confidence* (0.400), *high confidence* (0.700), and *highest confidence* (0.900). The version for the following analysis is version 9.1, and we only used the interactions above medium confidence since it is the default threshold of the STRING website. The results were produced using the *experiment* PPI score to avoid artificial correlation due to special interest in the research community.

#### HitPredict

The HitPredict database [39] provides the reliability scores of experimentally identified, physical *protein-protein interactions* (PPI). The database integrates five popular source database: IntAct, BioGrid, HPRD, DIP, and MINT. HitPredict also annotates the confidence scores into two levels: *low* and *high*. The version for the following analysis is version 4, and we only retrieved the interactions annotated with *high* quality.

### A knowledge-based *T*^2^-statistic

#### Data processing and pathway mapping

The required input format is a list of protein identifiers (e.g. UniProt accession number) with the expression ratios of the experimental group to the control. The list is subjected to the following data processing steps. First, we perform log_2_ transformation and winsorization on the data subsequently (if needed). Extreme values beyond the threshold are replaced with a maximum permitted value within the threshold. For example, if the expression ratios are (−7, −1, 3, 4, 6) and the threshold is set to 5, then the extreme value 6 will be replaced by the maximum permitted value 4, and -7 replaced by -4. Users can adjust the threshold based on the experimental conditions (i.e., different models or settings of mass spectrometry machines). The reason to set a threshold is to manually control the contribution of proteins with extreme values, since those proteins usually have very a low abundance (maybe beneath the threshold of quantification) in one of the experimental conditions. The default setting of the threshold is 10 and none of the testing data presented in this paper exceed the threshold. Second, we deal with the problem of multiple identifications and multiple ratios. This problem originates from the mapping of different primary identifiers to UniProt accession numbers. If there exists any ratio with multiple identifiers, we assign the same expression ratio to those identifiers. If there exists any identifier with multiple ratios, we assign the median of the ratios to the identifier. Third, we standardize the data by dividing the standard deviation. Last, we map these proteins with their expression ratios to the pathways. The identifier mapping tables are downloaded from the KEGG and the Ensembl [40] official websites.

#### The proposed *T*^2^-statistic

Hotelling’s *T*^2^-statistic is a multivariate generalization of Student’s *t* statistic. Let ***x***_1_, …, ***x***_*n*_ be *n* independent random variables of an *m*-variate normal distribution, an original Hotelling’s *T*^2^-statistic for an one-sample *T*^2^ test is defined as follows:

$$\begin{array}{c} T^{2}=n{\left(\bar{x}-\mu_{0}\right)}^{T}S^{-1}\left(\bar{x}-\mu_{0}\right) \end{array}$$

where $\bar{x}$ is the vector of column means, ***S*** is an *m* × *m* sample covariance matrix, and ***μ***_0_ is a given vector. The null hypothesis is that the population mean vector of data ***μ*** is equal to a given vector ***μ***_0_; $\bar{x}$ is served as an estimation of ***μ***.

Here we introduce the notation to describe the design of the proposed *T*^2^-statistic. Let a pathway P be the set of the proteins that are mapped from the data. Then,

$$\begin{array}{c} P=\left\{p_{i}|i=1,\ldots ,q\right\} \end{array}$$

where each *p*_*i*_ indicates a specific protein with a corresponding ratio *x*_*i*_, and *q* the number of mapped proteins (i.e., the size of a pathway). To ensure the biological robustness of the expression ratios {*x*_*i*_}, we have another threshold representing our confidence toward the expression direction of the data, the default setting is 1.5. Only the proteins showing more fold change beyond the threshold are qualified to possess their original ratios; the ratios of other proteins are recognized as disturbance and set to zero. We use the proteins with high expression ratios to represent the expression directions of the pathway. If directions of these proteins fit the covariance matrix, then the pathway is more likely to be enriched. In other words, we think the direction is not stable for those proteins of low expression ratios; we only care if the proteins of high expression ratios fit the covariance matrix. Again, users should adjust the threshold based on the experimental conditions.

We collect the processed ratios into a column vector ***x***. The proposed *T*^2^-statistic is constructed with the vector ***x*** and the covariance matrix ***S*** of ***x***, the former represents the significance of protein expression; the latter represents the interaction structure among the proteins. The covariance matrix ***S*** is not computed from the experimental data. Due to the limitation of sample size, calculating an informative covariance is infeasible. Our idea is to use the confidence score provided by protein-protein interaction databases to represent the strength of the covariance, and use the expression direction provided by the testing dataset to indicate the sign of the covariance. In order to apply this idea, we have an assumption below:

*If p_i_ and p_j_ have a strong interaction supported by experimental evidence, their expression ratios x_i_ and x_j_ will have a high covariance s_ij_ with a consistent sign*,

where *x*_*i*_ and *x*_*j*_ are any two elements of ***x*** and *s*_*ij*_ is the corresponding covariance of ***S***. To be more precise, we denote the PPI database as the set I and the interaction pairs collected as its elements:

$$I=\left\{c_{p_{v}p_{u}}|\begin{array}{l} p_{v}\text{ and }p_{u}\text{ are any two distinctive proteins with a confidence score}\\ c_{p_{v}p_{u}}\text{ describing the strength of their interaction}. \end{array}\right\}$$

On the basis of the confidence scores in I and the protein expression ratios, each element *s*_*ij*_ of ***S*** is determined by the following four rules:

1. For the elements where *i* = *j*, the main diagonal of ***S***, namely the variance of ***x***. In this case, there is no evidence score to refer, we have to assign a constant to the diagonal elements. This constant represents our confidence toward the accuracy of the data, we use the medium confidence level suggested by STRING, which is 0.4.
2. For the elements where *i* ≠ *j*, the confidence score between protein *p*_*i*_ and *p*_*j*_ exist in I, and the ratios *x*_*i*_ and *x*_*j*_ are of the same sign. In this case, either *x*_*i*_ and *x*_*j*_ are both up-regulated or both down-regulated, the covariance between them should be positive. We use *c*_*p*_*i*_*p*_*j*__ directly as the substitute of the covariance.
3. For the elements where *i* ≠ *j*, the confidence score between protein *p*_*i*_ and *p*_*j*_ exist in I, and the ratios *x*_*i*_ and *x*_*j*_ are of opposite signs. In this case, one of *x*_*i*_ and *x*_*j*_ is up-regulated and the other is down-regulated, the covariance between them should be negative. We take the negative value of *c*_*p*_*i*_*p*_*j*__ to be the substitute of the covariance.
4. For the elements where *i* ≠ *j*, and the confidence score between protein *p*_*i*_ and *p*_*j*_ does not exist in I. In this case, we assign zero to these elements.

The possible values of *s*_*ij*_ can be summarized as follows:

$$\begin{array}{c} s_{ij}=\left\{\begin{array}{cc} 0.4 & \text{if}\;i=j.\\ c_{p_{i}p_{j}} & \text{if}\;i\neq j,c_{p_{i}p_{j}}\in I,\;\text{and}\;x_{i}\cdot x_{j}\geq 0.\\ -c_{p_{i}p_{j}} & \text{if}\;i\neq j,c_{p_{i}p_{j}}\in I,\;\text{and}\;x_{i}\cdot x_{j}<0.\\ 0.0 & \text{if}\;i\neq j\;\text{and}\;c_{p_{i}p_{j}}\notin I. \end{array}\right. \end{array}$$

Since our aim is to test if a pathway P is differentially expressed, our null hypothesis can be described as follows:

*For those proteins mapped to the pathway being evaluated, they are not differentially expressed between distinctive phenotypes under certain structure of protein interaction*.

This self-contained null suggests that the expression ratios of data ***μ*** is equal to zero (i.e. the experiment and the control have the same expression values) under the same ***S***. We presuppose the sample size is equal to one because in data processing section we already use the median of ratios to represent the expression level for each protein. Therefore we use ***x*** as an estimator of ***μ***. The proposed *T*^2^-statistic for a specific pathway P is then defined as,

$$\begin{array}{c} T^{2}=x^{T}S^{-1}x∼\chi_{q}^{2} \end{array}$$

where ***x*** is the vector of expression ratios, ***x***^*T*^ is the transpose of ***x***, ***S***^−1^ is the inverse of the covariance matrix ***S***, and *q* is the number of mapped proteins in P. Since ***S*** is constructed from databases, it is possible that ***S*** is degenerate. In this case, we construct a Moore-Penrose pseudoinverse of ***S*** as a substitute, and *q* becomes the rank of ***S***. The *p*-value of the pathway P is derived from the $\chi_{q}^{2}$ distribution. Note that the *p*-values under a self-contained null are not for comparison among pathways. The significance only indicates if the pathway being tested, as an individual, is differentially expressed from one phenotype to another.

### Pathway integration

**Fig 1:**
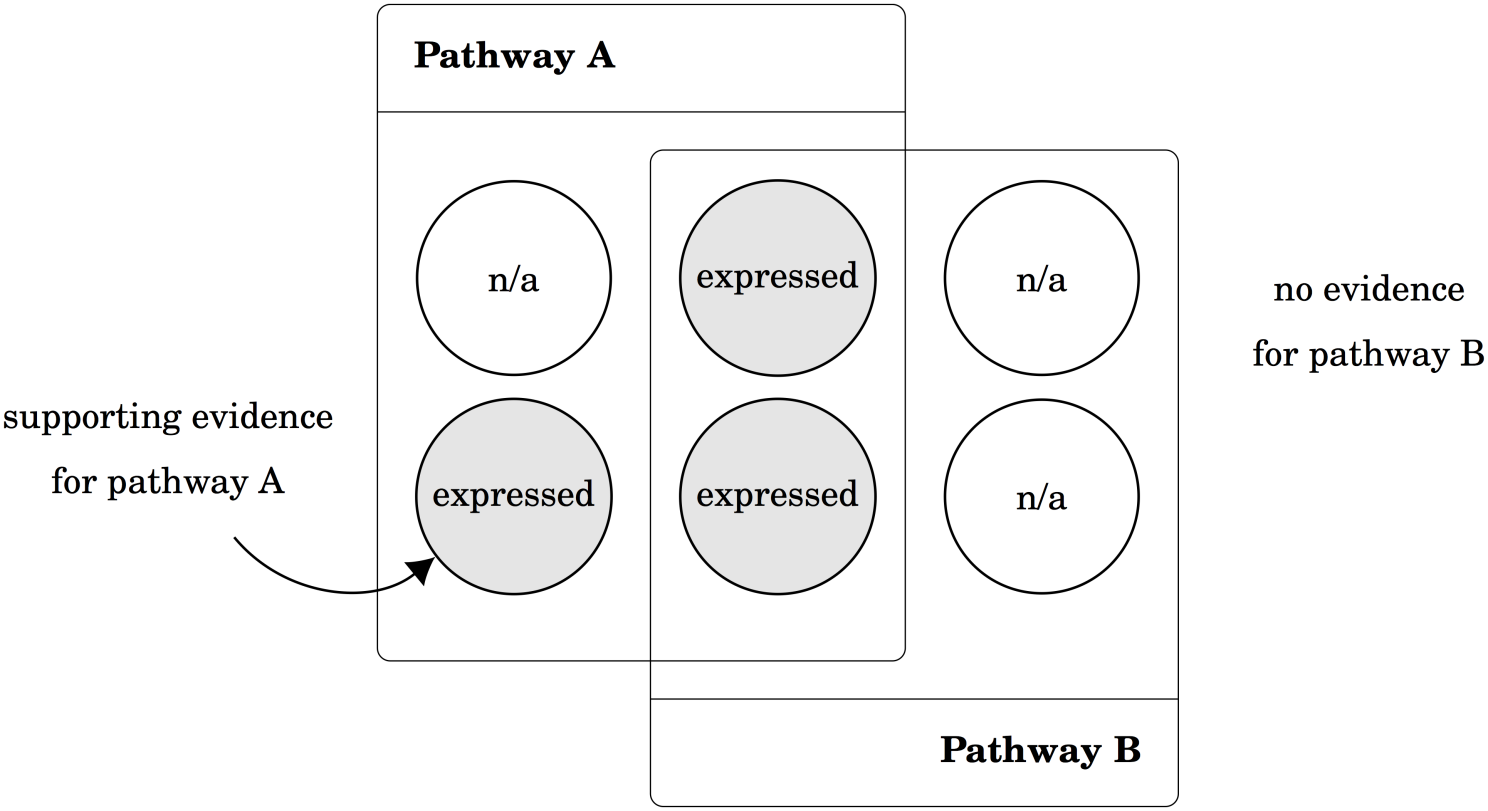
Redundant pathway versus non-redundant pathway. The expressed proteins of the pathway B are a subset of the expressed proteins of the pathway A. There is no evidence that pathway B is regulated since the significance may come from the merit of pathway A. On the contrary, some expressed proteins are unique to pathway A to support that pathway A may be regulated as a pathway group.

As we mentioned in Introduction, subsets of one common active module may cause a lot of pathways statistically significant. These pathways may only have slight relevance to the target mechanisms (Fig 1). To avoid misinterpretation due to irrelevant pathways being reported, we identify *delegates* to categorize pathways into *pathway groups*. A pathway group is a set of pathways, in which the pathway being the superset of other pathways is defined as the delegate. Since other pathways do not show any distinctive proteins to support themselves, the significance of other pathways may simply originate from the regulation of the delegate. In other words, we want to avoid the situation that a pathway is enriched only because of some common proteins that are shared with other pathways.

Our pathway integration procedure operates as follows. The set of the pathways being evaluated is denoted by P and each pathway P∈P is associated with a *p*-value. We iteratively perform the following steps until all the pathways in P are assigned to a specific pathway group M.

1. We identify the pathway with the maximum number of mapped proteins $P_{m}$ from P.
2. We create a new set $M=\{P_{m}\}$ for the delegate $P_{m}$, then we remove $P_{m}$ from P.
3. For each pathway P∈P, if $P\subseteq P_{m}$, we put P in M and remove P from P.
4. We check if there is any pathway remaining in P. If P≠⌀, we go back to the step 1. Otherwise, we check the *p*-value for every delegate $P_{m}$. Only those $P_{m}$ with a significant *p*-value are preserved as our final result.

A demonstrative example can be found in S1 Fig.

The pathway integration procedure aims to find the pathways with the most sufficient information to represent current data. Please notice that this idea is not similar to pathway hierarchy in which higher level pathways are defined as the supersets over lower level pathways, in that case only the pathways of highest level are able to be delegates. One can only apply the integration procedure to pathways without hierarchy relationship. In addition, the procedure is only applicable to the statistical test using a self-contained null since the measure of significance is independent for each pathway.

## Results and discussion

To demonstrate the advantages of the proposed *T*^2^-statistic, we compared our results with popular pathway analysis services within the community: Ingenuity^®^ Pathway Analysis (IPA), the Database for Annotation, Visualization and Integrated Discovery (DAVID), Gene Set Enrichment Analysis (GSEA), and Direction Pathway Analysis (DPA). Among these tools, IPA and DAVID use a competitive null; DPA uses a self-contained null; and GSEA uses a hybrid approach by default but suggests to use a gene sampling model when the sample size is smaller than seven [41]. The parameter settings for each tool are described in S1 Text. Their default significance requirements and ranking statistics are listed in Table 1. All of these tools apply univariate statistics, which means they do not consider the associations among gene products. We do not compare to any other multivariate statistics because using a multivariate statistics requires large sample size to calculate the covariance matrix, so they are not applicable to the proteomic data of only one experiment. In addition, the performance of *T*^2^-statistic has been evaluated in [17], which suggested that *T*^2^-statistic generally outperforms other tools in all simulation cases in their study. The proposed *T*^2^-statistic, DPA, and GSEA were evaluated using the same version of KEGG and Reactome databases; whereas DAVID had their own versions as web services and IPA was bounded to its own curated database. The following discussion mainly focused on the results of KEGG pathways since the they are canonical to the community; the results of Reactome pathways were summarize in S1 Table.

**Table 1:** The significance requirement and the ranking statistic for each tool.

Tools	Significance requirement	Ranking statistic
*T*^2^	raw *p*-value ≤ 0.05	number of mapped proteins
DPA	raw *p*-value ≤ 0.05	*p*-value
GSEA	FDR adjusted *p*-value (i.e. *q*-value) ≤ 0.25	NES
DAVID	EASE ≤ 0.1	*p*-value
IPA	Benjamini corrected *p*-value ≤ 0.05	*p*-value

The significance requirements and the ranking statistics for each tool are suggested in their associated publications or websites. For the pathways fulfilled the requirement of significance, we rank them using the ranking statistic.

As we mentioned above, different approaches of pathway analysis may have different statistical assumptions; comparing the performance between these approaches becomes even harder. Since there are no accepted gold-standards to evaluate the methods of pathway analysis, we tried to match the results reported by these methods to the biological idea provided by the original publication. In other words, we took the aspect of explanatory power (i.e. the interpretation of true positive cases). Generally speaking, a good statistic should be able to determine if a pathway is significant: if a statistic reports very little significant pathways (much less than our expectation regarding the data), it might have the problem of false negatives; if a statistic reports a large number of significant pathways, it might have the problem of false positive. Practically the false positive under a competitive null may originate from high mapping rate with low expression level; the false positive under a self-contained null may originate from low mapping rate with high expression level. We supposed that the number of significant pathways is one of the attribute to evaluate these methods, at least for the significance evaluation part (i.e. revealing the consequence of different null hypotheses). Also, a good statistic should be capable of enriching the true positive pathways. Based on these assumptions we had a preliminary evaluation as below.

**Fig 2:**
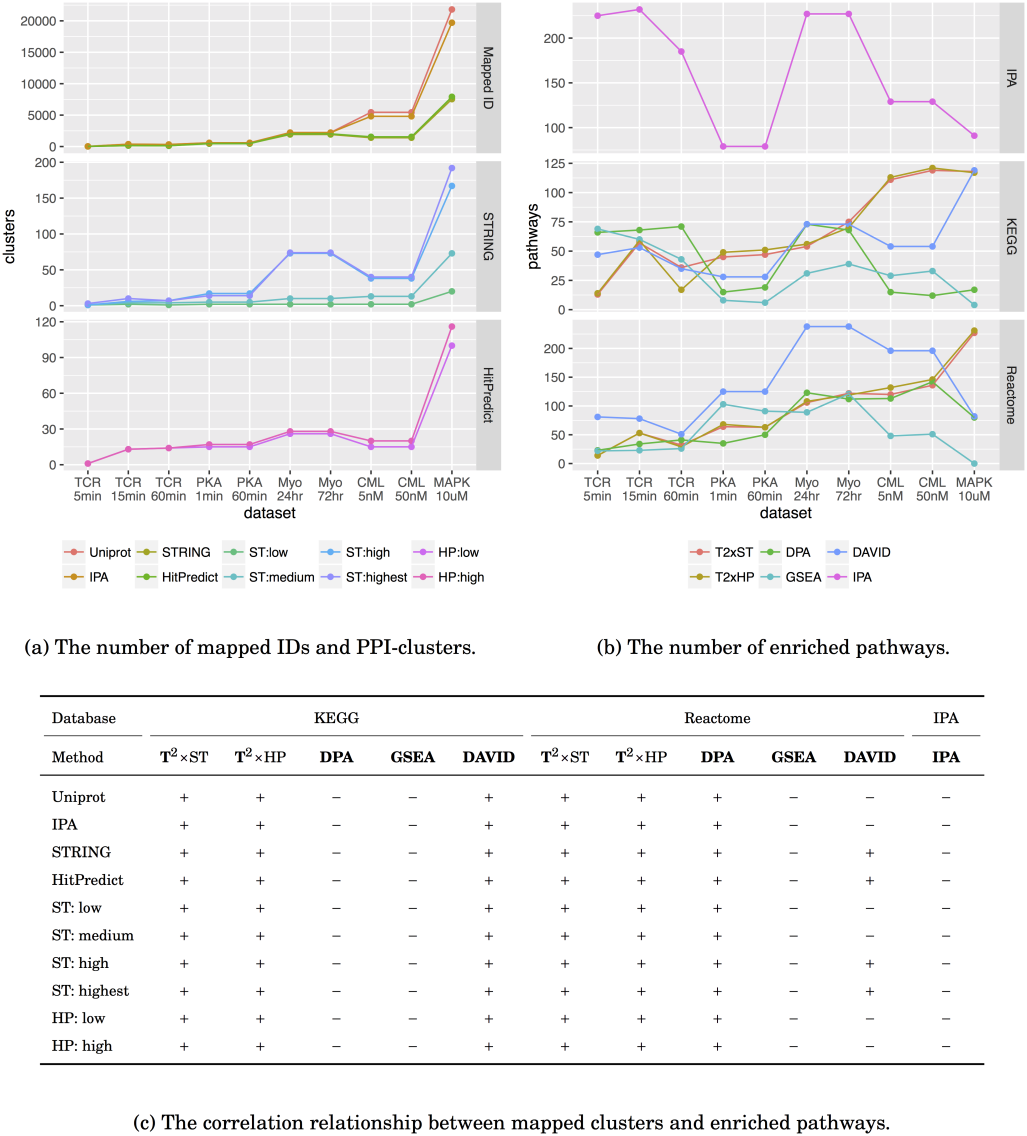
The consistency between data complexity and the number of enriched pathways. (a) The line chart indicated that the number of mapped IDs and the number of PPI-clusters have a close similarity. (b) The line chart showed an increasing trend of the number of enriched pathways for both *T*^2^ approaches; whereas the same trend can be hardly observed for other tools. (c) We compute the correlation coefficient between the number of mapped clusters and the number of enriched pathways. A plus sign (“+”) indicates a positive coefficient and a minus sign (“−”) indicates a negative coefficient.

The numbers of proteins, PPI clusters (i.e. the networks constructed by mapped proteins and interactions), and pathways for each dataset are listed in S2 Table, their trends and correlation are illustrated in Fig 2. With the exception of the TCR dataset, the identified proteins for each data in the same dataset are almost identical, yet with different amount of quantification values; the pathways enriched in the same dataset are similar. The numbers of enriched pathways should be related to the number of quantified proteins; and the number of potential treatment targets should also reveal the complexity of the five datasets. The treatment of the TCR dataset, anti-CD3*ϵ*, is a monoclonal antibody, whose target is specific and the influence should be limited. The case of the MAPK dataset is similar, U0126 is a highly selective inhibitor for MEK1 and MEK2. In the case of the PKA dataset, four targets of PGE2 are all subtypes of EP receptors (i.e. EP1, EP2, EP3, and EP4). Most of the downstream pathways are regulated by the second messenger cyclic adenosine monophosphate (cAMP) [31, 42]. On the other hand, dasatinib has about 10 targets of different kinase families [43] although its main target is BCR-ABL. The myogenesis dataset does not have a clear target, the size of the dataset is between the PKA dataset and the CML dataset. More targets suggest more proteins and pathways could be involved because of the treatment, and the number of enriched pathways should grow as well. Except for the gene expression data, we indeed observed an increasing trend of quantified proteins and PPI clusters (Fig 2a). However, the results of these tools (Fig 2b) do not totally concur with this hypothetical expectation. From Fig 2c we found both *T*^2^-statistics with positive coefficients between the data and the result, DPA and DAVID with some positive and some negative coefficients, and GSEA and IPA with negative coefficients all along. Having a negative coefficient between the data and the result is counter-intuitive, but a competitive null tends to behave in this fashion (GSEA in KEGG, GSEA and DAVID in Reactome, and IPA). Besides the null hypothesis, the construction of the statistics may also contribute. Both DPA and GSEA are based on the approach combining many gene-level statistics into a pathway-level statistics. The pathway-level statistic usually take into consideration the number of gene-level statistics it integrates, therefore the effect size for each gene-level statistic becomes important. In simple words, the average expression of the pathway dominates the enrichment result. For example, the TCR dataset is a cleansed dataset (S2a Fig) showed that there are only few proteins are of low expression ratios), almost all proteins are of high expression ratios. In this case, pathways can easily become significant because the average of expression ratios is usually high. In contrast, the PKA, the myogenesis, the CML, and the MAPK datasets have substantial proteins of low expression ratios (S2b, S2c, S2d and S2e Fig). These proteins will dilute the significance of others therefore the pathways can hardly become significant. Both DAVID and IPA are based on Fisher’s exact test, a small dataset will result unequally distribution among the cells of the table and pathways can easily become significant consequently. DAVID performs better because it uses a more stringent *post hoc* correction toward its significance level.

We determined the *target pathway* for each dataset according to their original publication: “TCR signaling pathway” for the TCR dataset, “cAMP signaling pathway” for the PKA dataset, “ECM-receptor interaction” for the myogenesis dataset, “chronic myeloid leukemia” for the CML dataset, and “MAPK signaling pathway” for the MAPK dataset. Generally the target pathway is directly provoked by the treatment, but in the myogenesis dataset, there does not exist such a clear target since the removal of serum takes away various kinds of growth factors. Therefore we chose one pathway that is clearly stated to be differentially expressed for both experimental conditions (24hr/0hr and 72hr/0hr). As a preliminary evaluation of performance, we tried to locate the target pathway among the significant pathways reported by each tool. The ranks of the target pathways were summarized in Table 2. Generally speaking, *T*^2^ with STRING (*T*^2^×ST) and *T*^2^ with HitPredict (*T*^2^×HP) found all the target pathways significant; and DAVID found eight out of ten; IPA found seven targets and DPA also found six. GSEA did not perform very well; it might because the design of the statistics is more suitable for the dataset of more experiments.

**Table 2:** The ranks of the target pathways in KEGG.

Dataset	TCR			PKA		Myogenesis		CML		MAPK
Treatment	*α*-CD3*ϵ*			PGE2		Serum-free		Dasatinib		U0126
	*5 min*	*15 min*	*60 min*	*1 min*	*60 min*	*24 hr*	*72 hr*	*5 nM*	*50 nM*	*10 μM*
KEGG pathway	T cell receptor signaling pathway			cAMP signaling pathway		ECM-receptor interaction		Chronic myeloid leukemia		MAPK signaling pathway
*T*^2^×ST *p*-value	1/13 < 0.0001	1/57 < 0.0001	2/36 0.0019	16/45 < 0.0001	17/47 < 0.0001	15/54 < 0.0001	16/75 < 0.0001	20/111 < 0.0001	23/119 < 0.0001	3/118 < 0.0001
*T*^2^×HP *p*-value	1/14 < 0.0001	1/59 0.0002	2/17 0.0011	17/49 < 0.0001	18/51 < 0.0001	16/56 < 0.0001	15/70 < 0.0001	20/113 < 0.0001	25/121 < 0.0001	3/117 < 0.0001
DPA *p*-value	-	8/68 0.0007	20/71 0.0024	11/15 0.0414	7/19 0.0178	18/73 0.0004	-	-	-	2/17 0.0007
GSEA *q*-value	68/69 0.1786	7/60 0.1039	-	-	-	22/31 0.1416	-	-	-	-
DAVID *p*-value	1/47 < 0.0001	1/53 < 0.0001	1/35 < 0.0001	-	-	40/73 0.0041	40/73 0.00041	8/54 0.0004	8/54 0.0004	8/119 0.0002
IPA *p*-value	1/225 < 0.0001	2/232 < 0.0001	89/185 0.0022	60/79 0.0186	60/79 0.0191	-	-	52/129 0.0013	52/129 0.0013	-

We had a preliminary evaluation of the pathway enrichment tools using the ranks of the target pathway for each testing dataset. The numerator is the rank value and the denominator is the number of significant pathways.

**Fig 3:**
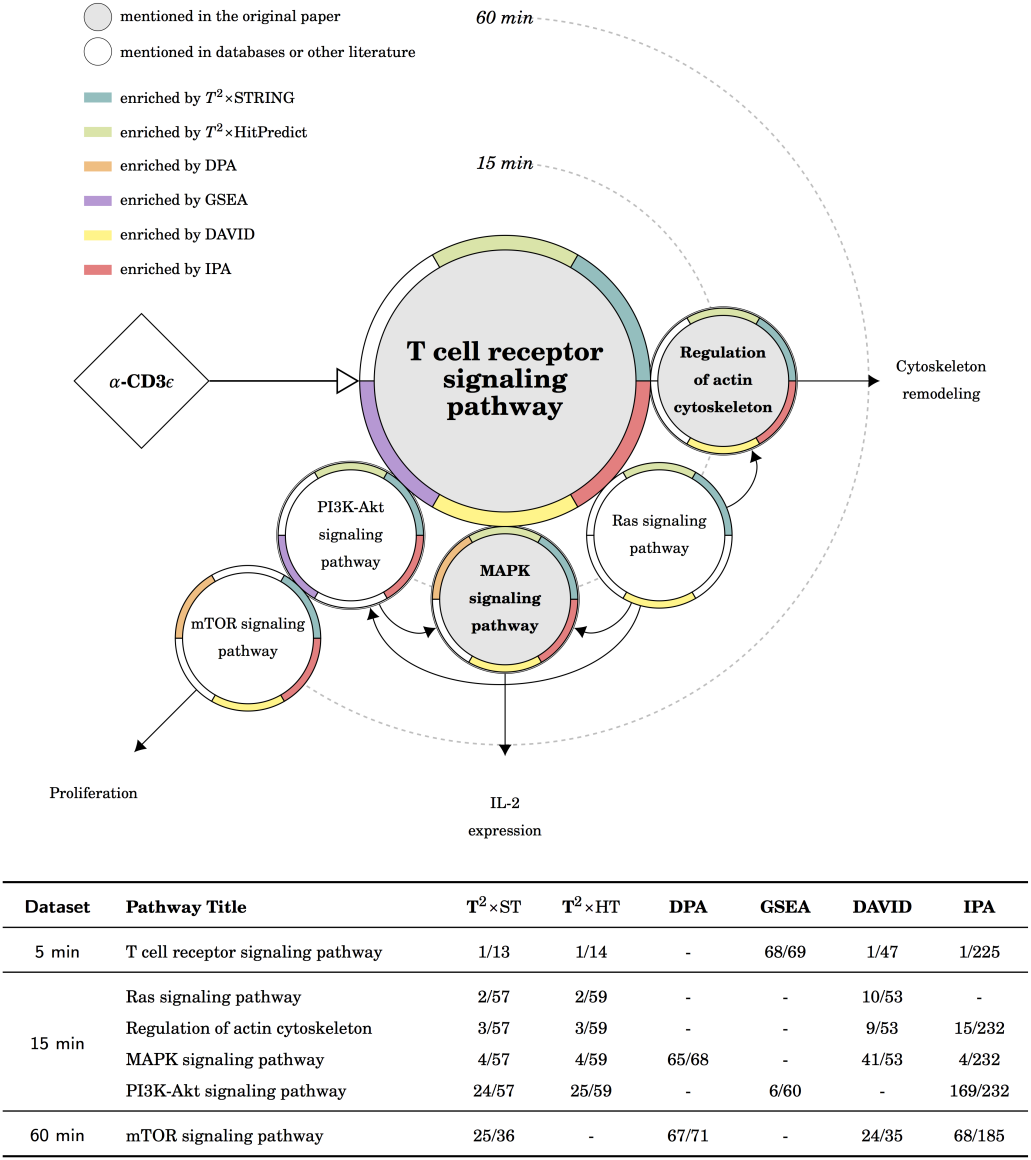
The results of the TCR dataset in KEGG. There are three possible downstream routes: the *IL-2 expression* and the *cytoskeleton remodeling* routes are the targets in the original publication, the former is enriched by *T*^2^×ST, *T*^2^×HP, DAVID, and IPA; the later is enriched by *T*^2^×ST, *T*^2^×HP, DAVID, and IPA; the *proliferation* route is also suggested by *T*^2^×ST and IPA.

**Fig 4:**
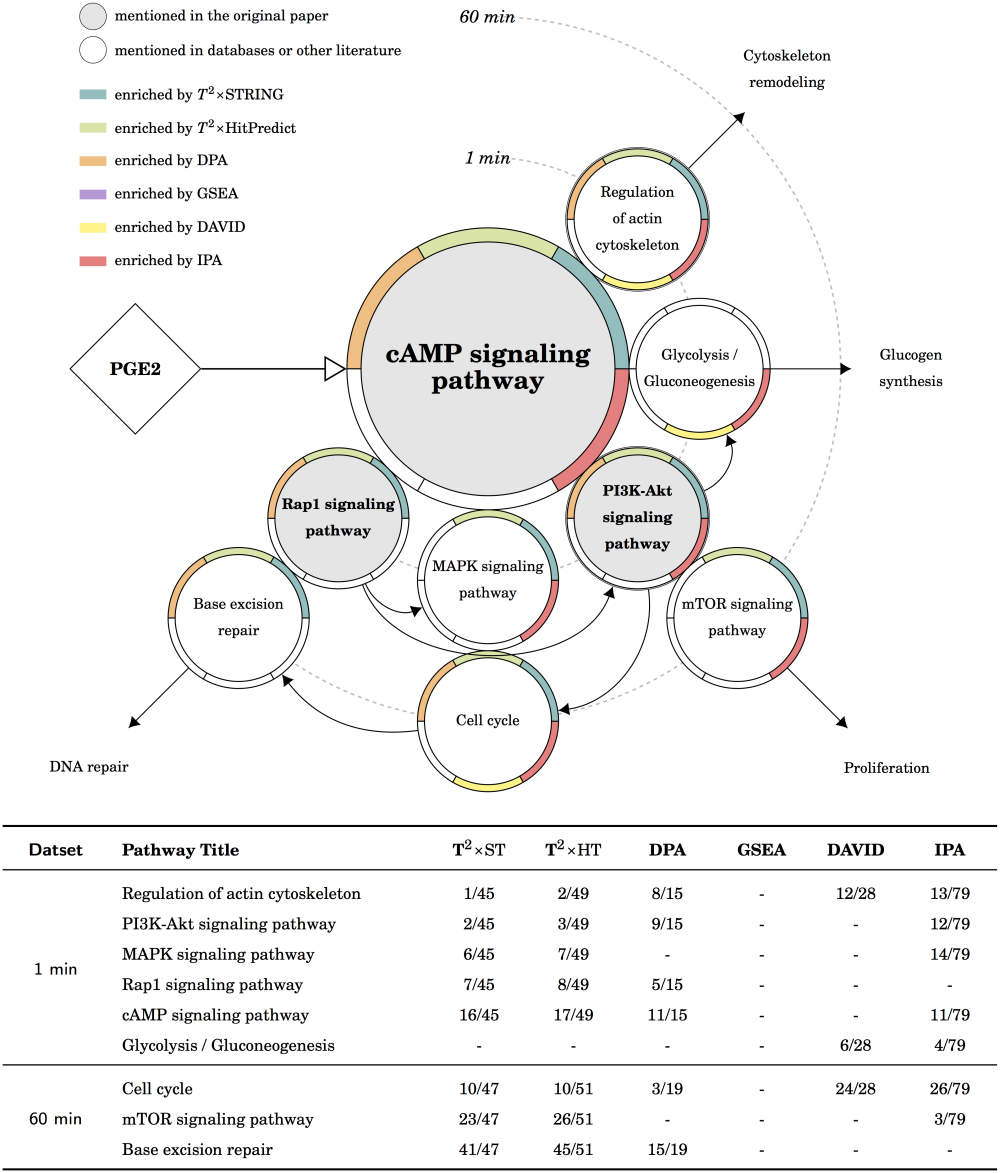
The results of the PKA dataset in KEGG. There are four possible downstream routes: the *proliferation* route is the target in the original publication and enriched by *T*^2^×ST, *T*^2^×HP, IPA; the *glycogen synthesis* route is also suggested by IPA; the *cytoskeleton remodeling* route is suggested by *T*^2^×ST, *T*^2^×HP, DPA and IPA; and the *DNA repair* route is suggested by *T*^2^×ST, *T*^2^×HP and DPA.

**Fig 5:**
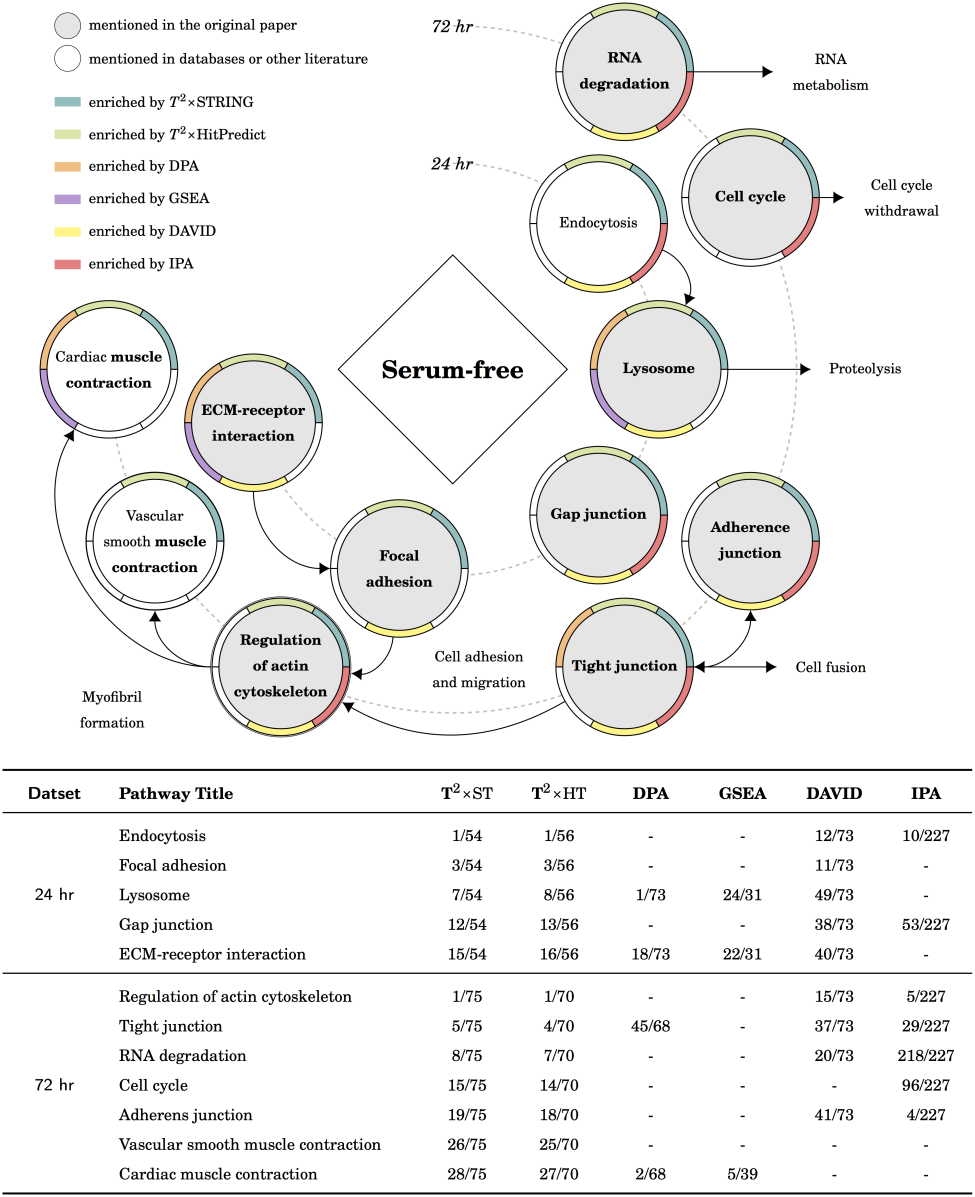
The results of the myogenesis dataset in KEGG. There are five routes mentioned in the original publication: the *RNA metabolism* route is enriched by *T*^2^×ST, *T*^2^×HP, DAVID and IPA; the *cell cycle withdrawal* route is enriched by *T*^2^×ST, *T*^2^×HP and IPA; the *proteolysis and cell fusion* route are suggested by *T*^2^×ST, *T*^2^×HP, and DAVID, and IPA; the *cell adhesion and migration* route are suggested by *T*^2^×ST, *T*^2^×HP and DAVID; and the *myofibril formation and muscle contraction* route is only enriched by *T*^2^×ST and *T*^2^×HP.

**Fig 6:**
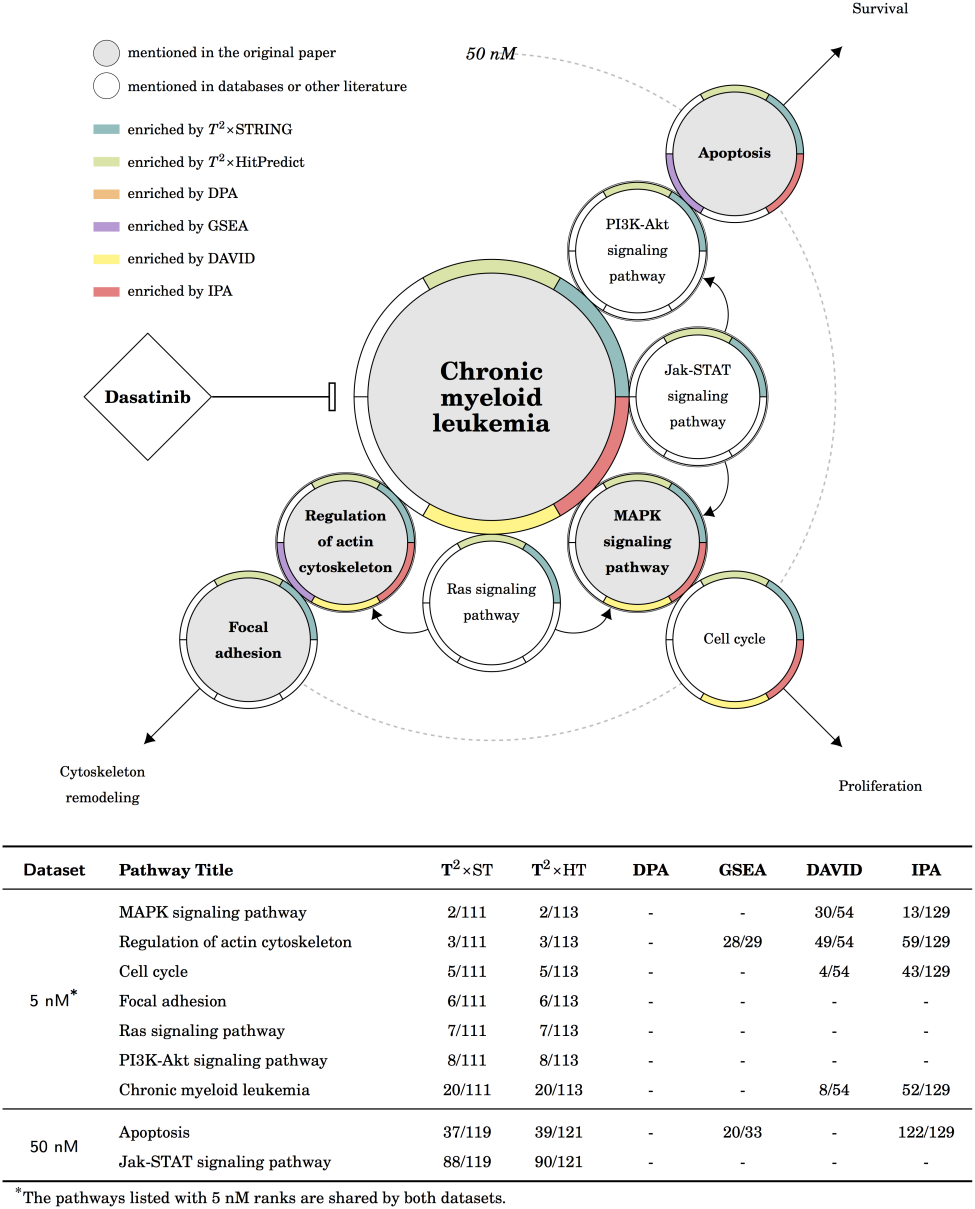
The results of the CML dataset in KEGG. There are three possible downstream routes: the *cytoskeleton remodeling* and the *survival* are the targets in the original publication, both of them are enriched by *T*^2^×ST and *T*^2^×HP; and the *proliferation* route is suggested by *T*^2^×ST, *T*^2^×HP, DAVID and IPA.

**Fig 7:**
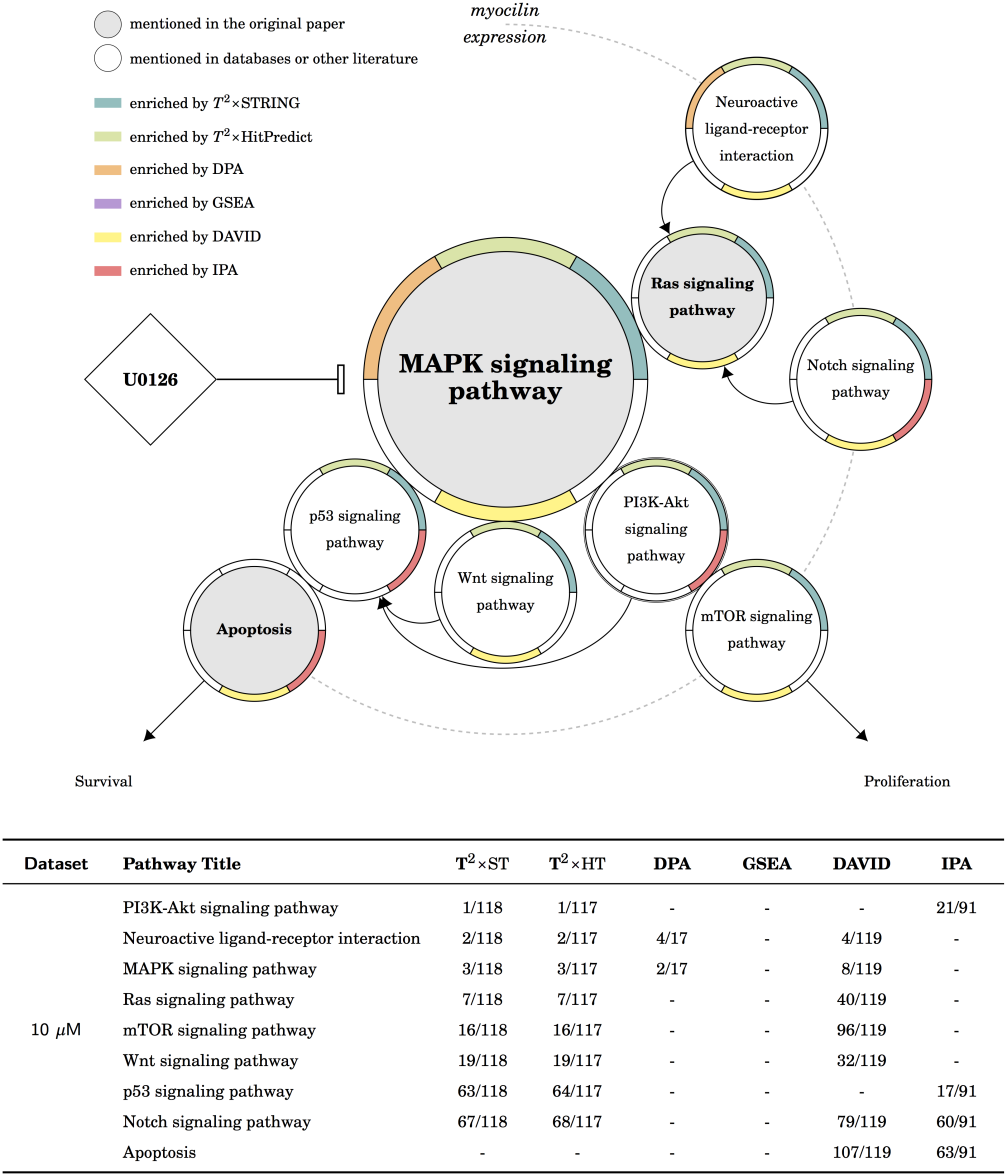
The results of the MAPK dataset in KEGG. There are two possible upstream routes: the *GPCR receptors* and the *notch receptors*. The former is enriched by *T*^2^×ST, *T*^2^×HP, DPA and DAVID; the later is enriched by *T*^2^×ST, *T*^2^×HP and DAVID. There are two possible downstream routes: both of the *proliferation* and the *survival* are the targets in the original publication. The former is enriched by *T*^2^×ST, *T*^2^×HP and DAVID; the later is roughly enriched by *T*^2^×ST, *T*^2^×HP, DAVID and IPA.

The mechanisms at pathway level behind the five datasets were depicted: Fig 3 for the TCR dataset, Fig 4 for the PKA dataset, Fig 5 for the myogenesis dataset, Fig 6 for the CML dataset, and Fig 7 for the MAPK dataset. Except for the myogenesis dataset, the target pathway is located in the center, related pathways are either neighbors or joined by arrows. Please note that the arrows between the treatment and the target pathway indicate the type of regulation (i.e. activation or inhibition) but the arrows between pathways indicate the direction of time. Each pathway is depicted by a circle with a ring of six segments: the background of a circle indicates the pathway is either the focus of the original publication or mentioned in pathway databases or literature; the color for each segment represents the significance reported by the certain tool. For example, in Fig 3, the T cell receptor signaling pathway is the focus of the original publication, and it is enriched by *T*^2^×ST, *T*^2^×HP, GSEA, DAVID, and IPA in the 5 min experiment. Please note that pathways are human-defined biological concepts, the components of one pathway may appear in other pathways and these components usually interact with each other. Our presentation only depicted the main branches directed from the target pathway that are commonly described in the literature. In addition, since the pathway database of IPA is different from others, we used the most similar pathway title instead. Even the pathway is of the same title, the components of the pathway may still differ; the result is obliquely comparable.

### The phosphoproteomic data of TCR signaling

We discussed the flow of signal transduction in time order since the dataset is a time-series of 5 min, 15 min, and 60 min experiments. In the beginning, the treatment anti-CD3*ϵ* activated TCR signaling pathway. The TCR signaling pathway in KEGG and IPA depicts the signals from the TCR receptors all the way to the IL-2 expression. Usually, the response of signal transduction comes rapid, we expected that the TCR signaling pathway should be enriched in early time points; the downstream from the TCR signaling pathway to the IL-2 expression should be enriched in late time points. From Table 2 we found that *T*^2^×ST, *T*^2^×HP, DAVID, and IPA enriched the TCR signaling pathway in all experiments; GSEA enriched the TCR signaling pathway in 5 and 15 min experiments only. The ranks of the TCR signaling pathway are top in *T*^2^, DAVID, and IPA. The downstream of the TCR signaling pathway was illustrated in Fig 3, there are three possible routes directed from TCR signaling. The original publication focused on the TCR/Ras/MAPK route to the IL-2 expression and the cytoskeleton remodeling response. The TCR/PI3K-Akt/mTOR route, on the other hand, is also important and has been discussed as a cluster in literature [44–47]. From Fig 3 we found the enriched pathways by *T*^2^×ST were well-grounded for the following reasons:

1. The phosphorylation of Ras/MAPK is an early event [30]. Most proteins in the Ras/MAPK route are phosphorylated at serine and threonine whereas the 5 min experiment only contains tyrosine-enriched peptides. As a result, related pathways should be enriched in the 15 min data, *T*^2^×ST, *T*^2^×HP, DAVID, and IPA achieved this goal.
2. Most proteins in the PI3K-Akt/mTOR are phosphorylated at serine and threonine, and the phosphorylation should happen in order. both *T*^2^×ST and IPA matched the description, but in IPA these pathways are of low ranks.
3. The regulation of actin as the response of TCR signaling is also a main subject of the original publication. *T*^2^×ST, *T*^2^×HP, DAVID, and IPA successfully enriched the cytoskeleton remodeling route.

To sum up above, we found the results of *T*^2^×ST fit our expectation: *T*^2^×ST enriched the TCR signaling pathway in the early time data, the route TCR/Ras/MAPK and TCR/PI3K-Akt/mTOR in time order, and the response of actin regulation, in both pathway databases. *T*^2^×HP, DAVID, and IPA enriched most of the expected pathways in KEGG, although the ranks of these pathways are occasionally low in IPA; *T*^2^×ST, *T*^2^×HP and DAVID also enriched most of the expected pathways in Reactome (S1 Table); GSEA failed to enrich half of the expected pathways in both pathway databases. We also found that there is no distinguishable difference between the result using a self-contained null (*T*^2^×ST, *T*^2^×HP, DPA) or a competitive null (GSEA, DAVID, IPA) in the TCR dataset.

### The phosphoproteomic data of cAMP/PKA signaling

The target of the original publication is PKA substrates. As the authors described in their results; PGE2 induced a rapid and maximal increase in phosphorylation level after 1 min, and the level remained high before the number of substrates gradually returned to near-basal conditions after 60 min (S2b Fig). From Table 2 we found *T*^2^×ST, *T*^2^×HP, DPA, and IPA enriched the cAMP signaling pathway for both 1 min and 60 min experiments. The downstream of the cAMP signaling pathway was illustrated in Fig 4, there are four possible routes directed from cAMP signaling. The proliferation route was suggested by *T*^2^×ST, *T*^2^×HP, and IPA; and the cytoskeleton remodeling route also, with an addition of DPA. The DNA repair route was enriched by *T*^2^×ST, *T*^2^×HP, and DPA, in both pathway databases. The glycogen synthesis route, on the other hand, was enriched only by IPA. The reason is that *T*^2^ takes expression ratios as an important feature, whereas the mapped proteins of the “Glycolysis / Gluconeogenesis” pathway are all of low ratios (min = −0.23710, max = 0.14950, mean = 0.02742).

Briefly, we found the results of *T*^2^ reasonable: *T*^2^×ST and *T*^2^×HP enriched the cAMP signaling pathway for both datasets; the PKA/Rap1/PI3K-Akt route in time order, and also the cytoskeleton remodeling route and the DNA repair route. DPA and IPA also enriched most of the expected pathways. GSEA and DAVID failed to enrich most of the expected pathways in both pathway databases. We also found that the result using a self-contained null (*T*^2^×ST, *T*^2^×HP, DPA) enriched more expected pathways than a competitive null (GSEA, DAVID, IPA). Since the pathways competes with each others under a competitive null, the success of some pathways will obstacle other pathways. For example, the top 1 enriched pathway provided by DAVID for the 1 min data is “Ribosome”. The pathway includes 87 proteins, and 51 of them were mapping by the data. The cAMP signaling pathway, on the other hand, includes 73 proteins, but only 8 of them were mapping by the data. The high mapping rate of “Ribosome” will makes it harder for DAVID to enrich the target cAMP signaling pathway.

### The cellular proteomic data of myogenesis

This study aims to characterize the changes in protein expression underlying the phenotype conversion from mononucleated muscle cells to multinucleated myotubes. According to the their analysis, five functional clusters were identified in this dataset: *cell cycle withdrawal* (72hr/0hr), *cell adhesion and migration* (24hr/0hr), *RNA metabolism* (both 24hr/0hr and 72hr/0hr), *myofibril formation* (72hr/0hr), and *proteolysis, fusion, and ECM remodeling* (both 24hr/0hr and 72hr/0hr). The corresponding KEGG pathways were illustrated in Fig 5. We chose the pathway “ECM-receptor interaction” as the target pathway in Table 2 because it is clearly stated to be differentially expressed in both experiments. From Table 2 we found the ECM-receptor interaction pathway was enriched by *T*^2^×ST, *T*^2^×HP and DAVID for both experiments. The original publication focused on the change of cellular phenotype accompanying myogenic differentiation and the development of myofibril. From Fig 5 we found the myofibril formation route was enriched by *T*^2^×ST, *T*^2^×HP, DAVID, and IPA. *T*^2^×ST and *T*^2^×HP further enriched muscle contraction related pathways, which were associated with myotube maturation as discussed in the original publication. The cell adhesion and migration play an essential role in the fusion of mononucleated myoblasts. Pathways related to adhesion and migration were enriched by *T*^2^×ST, *T*^2^×HP, and DAVID; those related to fusion were enriched by *T*^2^×ST, *T*^2^×HP, DAVID, and IPA. The elevation of lysosomal proteins contributed in remodeling intracellular components during the course of myotube formation. All tools enriched the lysosome pathway with an exception of IPA. The RNA metabolism and the cell cycle withdrawal routes represented the termination of proliferation since the growth factors and nutrition were removed from the medium. Related pathways were enriched by *T*^2^×ST, *T*^2^×HP and IPA. Briefly, *T*^2^×ST and *T*^2^×HP enriched all the pathways discussed in the original publication; the interpretation made by *T*^2^ fit the description of the dataset pretty well. In the meantime, DAVID and IPA also enriched most of the expected pathways, asides from muscle contraction pathways. DPA enriched most of the expected pathways in Reactome, but failed in KEGG; GSEA failed in both pathway databases. We also found that there is no distinguishable difference between the result using a self-contained null (*T*^2^×ST, *T*^2^×HP, DPA) or a competitive null (GSEA, DAVID, IPA) in the myogenesis dataset.

### The phosphoproteomic data of BCR-ABL signaling for CML treatment

This dataset, unlike the previous, is not a time series; it is a dose-comparison experiment. According to the original publication, two datasets shared nearly all identified proteins although 50nM dataset did down-regulate more phosphopeptides (S2d Fig). Consequently, the authors only paid attention to the proteins that are regulated by both 5nM and 50nM dasatinib. The main target of dasatinib is the BCR-ABL signaling pathway, which is described in the pathway “Chronic myeloid leukemia (CML)” of KEGG. From Table 2 we found the CML pathway was enriched by *T*^2^×ST, *T*^2^×HP, DAVID and IPA for both experiments. The downstream of the CML pathway was illustrated in Fig 6, there are three possible routes directed from the inhibition of BCR-ABL. Since the two datasets shared nearly all proteins, we used the ranks of 5 nM dataset to represent the common pathways over two datasets. The original publication focused on the BCR-ABL/Ras/MAPK route and the connection between BCR-ABL signaling and apoptosis. From Fig 6 we found the enriched pathways by *T*^2^ were plausible for the following reasons:

1. In the case of the BCR-ABL/Ras/MAPK route, *T*^2^×ST, *T*^2^×HP, DAVID, and IPA all agreed with the description. The BCR-ABL/PI3K-Akt/Apoptosis route was also enriched by *T*^2^×ST, *T*^2^×HP and IPA.
2. Even though not mentioned in the original publication, the BCR-ABL/JAK-STAT/Apoptosis route is also well-known [48–50]. Among all the tools for comparison, *T*^2^×ST and *T*^2^×HP were the only tools that enriched the “JAK-STAT signaling pathway” in 50nM data.
3. The initiation of focal adhesion components is also related to BCR-ABL. The STRING analyzing result of the original publication also implied the mechanisms of cytoskeleton remodeling. *T*^2^×ST, *T*^2^×HP, DAVID, and IPA achieved to enrich the pathway “Regulation of actin cytoskeleton”; but only *T*^2^×ST and *T*^2^×HP enriched the “Focal adhesion” pathway.

In short, we found the performance of *T*^2^ pleasant: *T*^2^×ST and *T*^2^×HP enriched the BCR-ABL/Ras/MAPK route, the BCR-ABL/PI3K-Akt/Apoptosis route, the JAK-STAT signaling pathway, and the pathways related to actin response. Both DAVID and IPA enriched some of the expected pathways. GSEA and DPA failed to enrich most of the expected pathways in both pathway databases. Generally there is no distinguishable difference between the result using a self-contained null or a competitive null, but *T*^2^ enriched more expected pathways than other methods.

### The gene expression data of MAPK signaling

The dataset includes only one experiment, comparing the gene expression of myocilin expressed cells to control cells, under U0126 treatment. The authors concluded that myocilin has a protective effect to against apoptosis and further promotes cell survival and proliferation via the MAPK signaling pathways. They also experimentally confirmed that the Raf-MEK-ERK-MAPK cascade was activated by myocilin. From Table 2 we found *T*^2^×ST, *T*^2^×HP, DPA, and DAVID enriched the MAPK signaling pathway; all three methods under a self-contained null successfully enriched the target pathway, whereas only DAVID is under a competitive null. The upstream and downstream of the MAPK pathway was illustrated in Fig 7. There are two possible upstream receptors of the MAPK signaling pathway: the GPCR receptors and the Notch receptors. In KEGG, both upstreams were enriched by *T*^2^×ST, *T*^2^×HP and DAVID, DPA only enriched the GPCR/Ras/MAPK route and IPA only enriched the Notch/Ras/MAPK route. In Reactome, both upstreams were enriched by *T*^2^×ST and *T*^2^×HP, DAVID only enriched the Notch/Ras/MAPK route (S1 Table). The are two downstream routes, both are supported by the original publication. The results from both *T*^2^×ST and *T*^2^×HP suggested that the differentially expressed pathways are more upstream. This conclusion actually fit the discussion of the original publication, which suggests that myocilin may also regulate the upstream kinase of the MAPK signaling pathway. Briefly, both *T*^2^×ST and *T*^2^×HP successfully enriched the MAPK signaling pathway and its upstream; other tools enriched only some of the expected pathways.

Generally speaking, we found that the proposed *T*^2^-statistic was able to enrich the pathways in agreement with the original publication, whereas the performances of DPA, GSEA, and DAVID were not stable. IPA, as a commercial software with high cost, also enriched most of the relevant pathways. Nevertheless some of the pathways are low-ranked and the numbers of enriched pathways are enormous. The results suggested that our multivariate design of the proposed *T*^2^-statistic does provide important information toward pathway analysis by considering the strength of interactions among proteins. In the meantime, our self-contained null hypothesis is capable of enrich relevant pathways by the significance of protein expression ratios, whereas the focus of the competition among pathways may neglect the clear distance between phenotypes. Both DAVID and IPA are based on the competitive null hypothesis, although DAVID performs a more stringent *post hoc* correction, they shared the failure of some relevant pathways. The tremendous numbers of enriched pathways also suggested IPA may report more false positive results. GSEA also applies a competitive null when the sample size is limited and its KS statistic is sensitive to small sample size. The unsatisfied results suggested that GSEA is not suitable for data of limited experiments. The design of DPA is similar to the proposed *T*^2^-statistic; both DPA and *T*^2^ are specifically designed for quantitative proteomic data, and they both use self-contained null hypotheses. The performance difference between DPA and *T*^2^ primarily comes from the aspect of statistic construction. The proposed *T*^2^-statistic outperformed DPA because it considers the strength of interactions among proteins. Briefly, in five testing datasets, the results using a self-contained null is generally more well founded than the results using a competitive null.

### Robustness test: Using permuted and purged confidence scores

**Fig 8:**
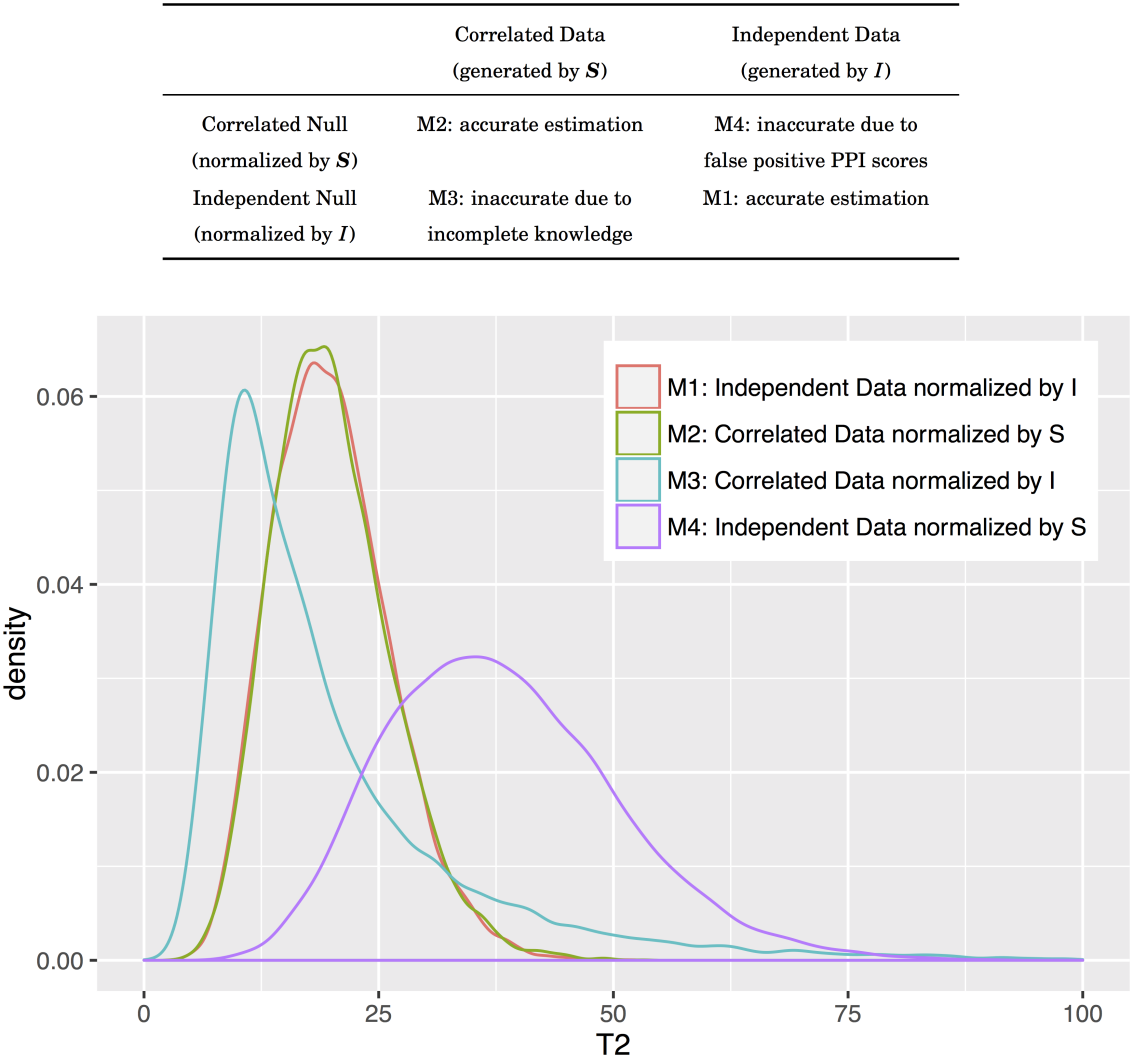
A demonstrative example of accurate and inaccurate estimation. We simulated a toy example to demonstrate the situation that may cause inaccurate estimation. The simulation data consisted 10000 random samples; the correlated data are derived from MVN (0, ***S***) and the independent data are from MVN(0, *I*), where ***S*** is a 20 × 20 matrix with diagonal equals to one and off-diagonal equals to 0.5.

The importance of applying the covariance matrix is to estimate accurate confidence interval. We illustrated an example in Fig 8 to demonstrate the situation that may cause inaccurate estimation. Both M1 and M2 are accurate null distributions since the data are normalized using proper covariance matrix and the distribution hence follows *χ*^2^. M4 indicates that situation that an independent data are misinterpreted as a correlated data. This happens when we have false positive protein-protein interactions in the databases. In order to minimize the risk, we only use the confidence scores derived from directly experimental evidence. M3 represents the case that a correlated data are misinterpreted as an independent data. This may actually happen due to our incomplete knowledge of the biology system. In this case, the null distribution will not follow *χ*^2^ and the estimation of *p*-value will be inaccurate. Even so, for pathways of vary high expression ratios, applying the covariance matrix or not does not change its *p*-value dramatically. Here we demonstrated the impact toward *p*-values using permuted and purged confidence scores. We performed 100 experiments with 30% and 60% permuted confidence scores (i.e. we reassign the score using the same score distribution) and another 100 experiments with 30% and 60% purged confidence scores (i.e. we randomly remove the scores from the PPI databases), and we checked if the expected pathways are still significant under current significance level (*α* = 0.05). From Table 3 we observed that:

1. In practice, most of expected pathways contain proteins of high expression ratios, so the influence toward their *p*-values is limited.
2. Some pathways are more easily to be affected by permuted scores rather than purged scores, such as *Cell cycle* in CML 5 nM experiment and *PI3K-Akt signaling pathway* in MAPK 10 *μ*M experiment. In this case, the covariance matrix for the pathway is close to an identity matrix. It might be a M3 case that we do not have enough information to construct the covariance matrix, the *p*-values tend to be smaller, so the interpretation of significant pathways should be careful.
3. Some pathways are more easily to be affected by purged scores rather than permuted scores, such as *PI3K-Akt signaling pathway* in PKA 1 min experiment and *Endocytosis* in myogenesis 72 hr experiment. In this case, the covariance matrix for the pathway provides abundant information. The removal of these information will decrease the chance for the pathway to be enriched. It might be a M4 case that we use some fake information to construct the covariance matrix, the *p*-values tend to be larger, so the users should manually examine the pathways of borderline *p*-values.

**Table 3:** The results using permuted and purged confidence scores.

Dataset	Experiment	Pathway Title	original *p*-value	30% permuted	60% permuted	30% purged	60% purged
TCR	5 min	T cell receptor signaling pathway	< 0.0001	100%	100%	100%	100%
	15 min	Ras signaling pathway	0.0017	100%	100%	100%	100%
		Regulation of actin cytoskeleton	< 0.0001	100%	100%	100%	100%
		MAPK signaling pathway	< 0.0001	100%	100%	100%	100%
		PI3K-Akt signaling pathway	< 0.0001	100%	100%	100%	100%
	60 min	mTOR signaling pathway	0.0002	97%	98%	100%	100%
PKA	1 min	Regulation of actin cytoskeleton	< 0.0001	100%	100%	100%	100%
		PI3K-Akt signaling pathway	< 0.0001	92%	81%	86%	84%
		MAPK signaling pathway	< 0.0001	100%	100%	100%	100%
		Rap1 signaling pathway	< 0.0001	100%	100%	100%	100%
		cAMP signaling pathway	< 0.0001	100%	100%	100%	100%
		Glycolysis / Gluconeogenesis	1	0%	0%	0%	0%
	60 min	Cell cycle	< 0.0001	100%	100%	100%	100%
		mTOR signaling pathway	0.0024	100%	100%	100%	100%
		Base excision repair	< 0.0001	100%	100%	100%	100%
myogenesis	24 hr	Endocytosis	0.0441	60%	64%	48%	69%
		Focal adhesion	< 0.0001	100%	100%	100%	100%
		Lysosome	< 0.0001	100%	100%	100%	100%
		Gap junction	< 0.0001	100%	100%	100%	100%
		ECM-receptor interaction	< 0.0001	100%	100%	100%	100%
	72 hr	Regulation of actin cytoskeleton	< 0.0001	100%	100%	100%	100%
		Tight junction	< 0.0001	100%	100%	100%	100%
		RNA degradation	< 0.0001	100%	100%	100%	100%
		Cell cycle	< 0.0001	100%	99%	98%	91%
		Adherens junction	< 0.0001	100%	100%	100%	100%
		Vascular smooth muscle contraction	< 0.0001	100%	100%	100%	100%
		Cardiac muscle contraction	< 0.0001	100%	100%	100%	100%
CML	5 nM	MAPK signaling pathway	< 0.0001	100%	100%	100%	100%
		Regulation of actin cytoskeleton	< 0.0001	100%	100%	100%	100%
		Cell cycle	0.0001	90%	92%	98%	99%
		Focal adhesion	< 0.0001	100%	100%	100%	100%
		Ras signaling pathway	< 0.0001	100%	100%	100%	100%
		PI3K-Akt signaling pathway	< 0.0001	100%	100%	100%	100%
		Chronic myeloid leukemia	< 0.0001	100%	100%	100%	100%
	50 nM	Apoptosis	< 0.0001	100%	100%	100%	100%
		Jak-STAT signaling pathway	0.0003	100%	100%	100%	100%
MAPK	10 *μ*M	PI3K-Akt signaling pathway	0.0271	70%	69%	90%	90%
		Neuroactive ligand-receptor interaction	< 0.0001	100%	100%	100%	100%
		MAPK signaling pathway	< 0.0001	100%	100%	100%	100%
		Ras signaling pathway	< 0.0001	100%	100%	100%	100%
		mTOR signaling pathway	< 0.0001	100%	100%	100%	100%
		Wnt signaling pathway	0.001	100%	100%	100%	100%
		p53 signaling pathway	< 0.0001	100%	100%	100%	100%
		Notch signaling pathway	0.0252	82%	87%	94%	99%
		Apoptosis	0.9882	0%	0%	0%	0%

The percentage indicated the proportion that was successfully enriched under current permutation or removal setting. Pathways highlighted by green background are easily affected by permuted scores than purged scores; pathways highlighted by red background are easily affected by purged score than permuted scores.

The construction of the proposed *T*^2^-statistic showed *T*^2^ is heavily dependent on expression ratios. After all, the null hypothesis for *T*^2^ is to test if the mean vector equals to zero. The contribution of applying the covariance matrix is to estimate *p*-values in a more accurate manner: to rescue some pathways with moderate expression ratios but their regulation directions are consistent with current knowledge of protein interaction, and to discard some pathways with inconsistency.

## Conclusion

In this study, we presented a knowledge-based *T*^2^ approach to perform pathway analysis for quantitative proteomic data of a limited number of experiments. The proposed *T*^2^ is constructed as a multivariate statistic and the test of significance is under a self-contained null. We use the probabilistic confidence score provided by the STRING or HitPredict databases to approximate the covariance matrix of the protein profiles. The proposed *T*^2^-statistic is therefore able to reveal the influence of protein-protein interactions while performing the analysis. In addition, our pathway integration procedure is able to categorize pathways into pathway groups as well as to avoid redundancy. We performed the *T*^2^-statistic on five published quantitative proteomic dataset. In all cases, *T*^2^ was able to eliminate irrelevant pathways, as well as correctly identify relevant pathways that had been discussed in the original publication. The idea of incorporating biological evidence into conventional statistic can be widely applied to the analysis of quantitative proteomic data.

## Supporting information

S1 Text Versions and parameter settings of other tools. (PDF)

S1 Fig A demonstrative example of pathway integration procedure. This diagram used a toy example to illustrate the procedure of data processing, filtering, pathway mapping, statistical testing, and finally pathway integration.
(PDF)

S2 Fig Data distribution. (a) The TCR dataset contains three proteomic experiments. The 5 min data describe the initiation of the TCR signaling pathway. The following response interfered lots of downstream proteins, resulting the 15 min data have the largest number of proteins among this dataset. Then the signal was transmitted to the nuclear and the amount of high-ratio proteins decreased, as described in the 60 min data. (b) The PKA dataset contains two proteomic experiments. The initiation of the cAMP signaling pathway came rapid, so the 1 min data almost illustrate all the following events. The 60 min data have fewer high-ratio proteins because the response of the signal had gradually vanished. (c) The myogenesis dataset contains two proteomic experiments. The 24 hr data have more differentially expressed proteins than the 72 hr data. (d) The CML dataset contains two proteomic data. Their distributions look quite alike, despite that the 50 nM treatment did down-regulate more proteins. Most proteins are down-regulated since the experiment aims to repress the BCR-ABL signaling pathway. (e) The MAPK dataset contains one gene expression experiment. The up-regulated probes slightly outnumber the down-regulated (54% versus 46%), and there are few probes of high ratios.
(PDF)

S1 Table The results of all datasets in Reactome. (PDF)

S2 Table The number of proteins and pathways for each dataset. The enriched pathways are the pathways that fulfilled the significance requirements described in Table 1. We listed the number of clusters using different confidence levels. The number of PPI clusters is calculated for clusters with at least two proteins. Anti-CD3*ϵ* is specific to CD3*ϵ*; PGE2 targets four EP receptors but the downstream is almost under control of cAMP; serum starvation may result in cell cycle arrest and turn on muscle regulatory factors to promote myogenesis; dasatinib mainly targets BCR-ABL but has about 10 other targets of different kinase families; U0126 is a highly selective inhibitor of MEK1 and MEK2.
(PDF)
