# Supplementary material for: A knowledge-based T2-statistic to perform pathway analysis for quantitative proteomic data
Source: PLoS Comput Biol. 2017 Jun 16;13(6):e1005601. doi: 10.1371/journal.pcbi.1005601 (PMC5493430; doi:10.1371/journal.pcbi.1005601)
Supplement: S1 Text — (PDF) [file pcbi.1005601.s001.pdf]

A knowledge-based T^2-statistic to perform pathway analysis for quantitative proteomic data

En-Yu Lai[1,2], Yi-Hau Chen[3], and Kun-Pin Wu[1,*]

[1]*Institute of Biomedical Informatics, National Yang-Ming University, Taipei 11221, Taiwan.*

[2]*Bioinformatics Program, Taiwan International Graduate Program, Institute of Information Science, Academia Sinica, Taipei 11529, Taiwan.*

[3]*Institute of Statistical Science, Academia Sinica, Taipei 11529, Taiwan.*

Supplementary Method: Parameter setting of other tools

DPA We used the DPA R package v1.3. The testing data was applied on the same version of KEGG and Reactome databases, the parameter "minSize" was changed from 5 to 1; other settings remained default. To compare with other tools, the number of experiment was set to one constantly, and the data were not be rotated.

GSEA We downloaded the GSEA java application v2.2.3. The testing data was applied on the same version of KEGG and Reactome databases, we only changed the parameter "exclude minimum size" from 15 to 1; "permutation type" from "phenotype" to "gene set" according to the user guide, other settings remained default.

DAVID We use the DAVID web service v6.8. We applied the functional annotations on the testing data. The result was retrieved from the directory "KEGG" and "Reactome" under the directory "Pathways". All settings remained default.

IPA We applied the core analysis on the testing data. The result was retrieved from the list of canonical pathways. An additional threshold of fold change (FC$\geq$ 1) is applied on the MAPK dataset since the number of genes exceed 8000 (maximum accepted number of IPA); the threshold is recommended by the original paper of MAPK dataset. The accessed date is March 9, 2017 (IPA Winter Release).
