# Supplementary figures and images for: A knowledge-based T2-statistic to perform pathway analysis for quantitative proteomic data
Source: PLoS Comput Biol. 2017 Jun 16;13(6):e1005601. doi: 10.1371/journal.pcbi.1005601 (PMC5493430; doi:10.1371/journal.pcbi.1005601)

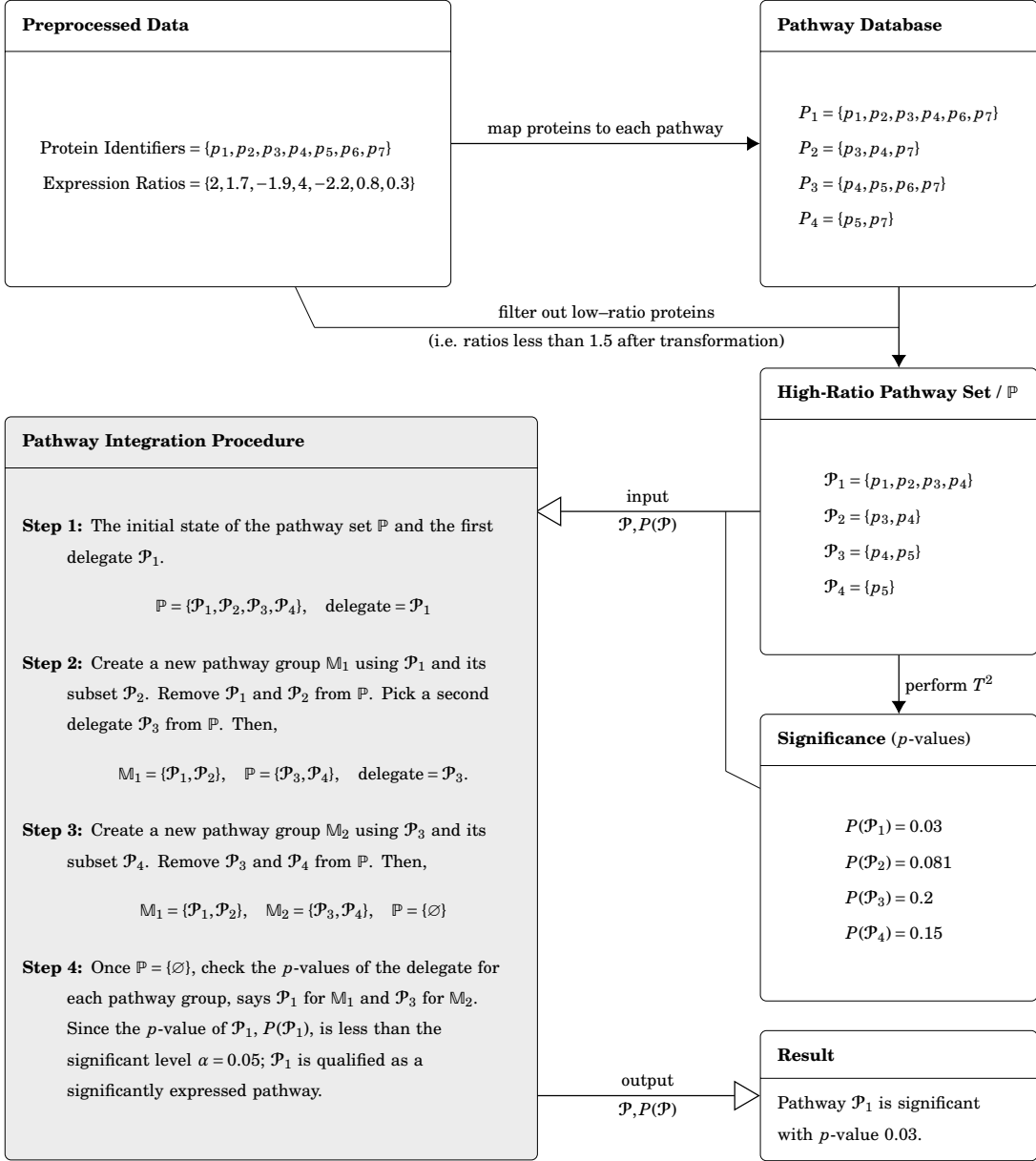

Supplement: S1 Fig — This diagram used a toy example to illustrate the procedure of data processing, filtering, pathway mapping, statistical testing, and finally pathway integration. (PDF) [file pcbi.1005601.s002.pdf]

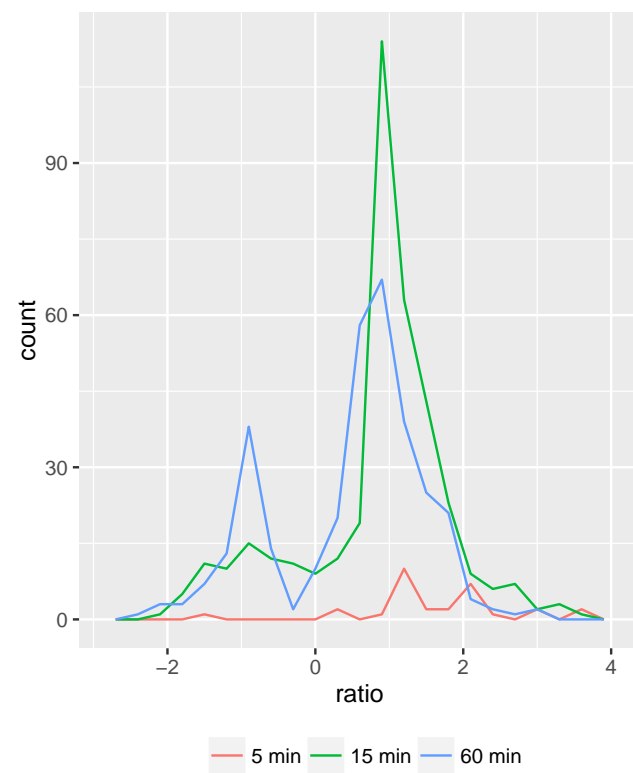

(a) TCR dataset.

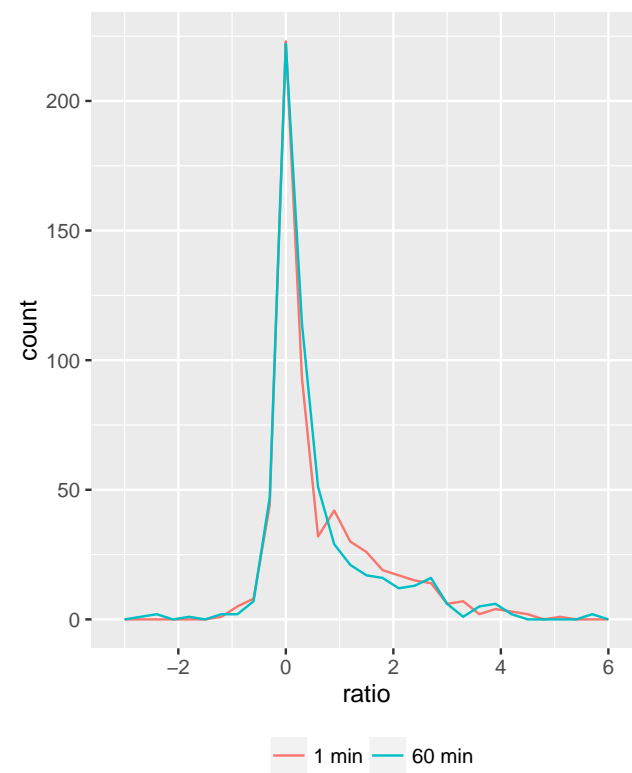

(b) PKA dataset.

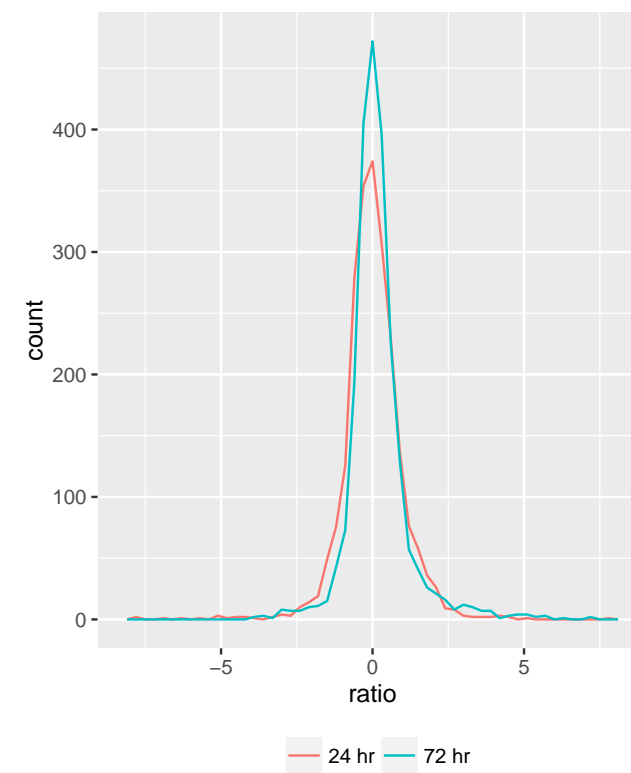

(c) Myogenesis dataset.

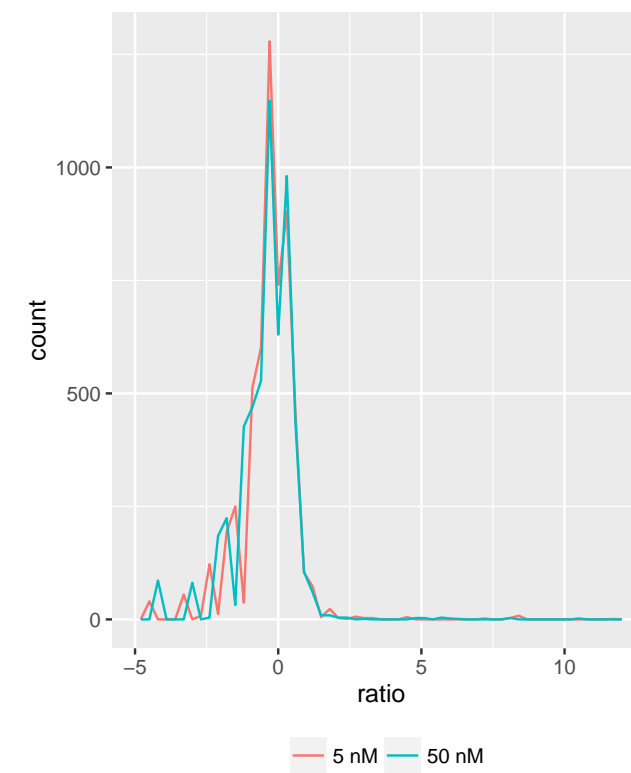

(d) CML dataset.

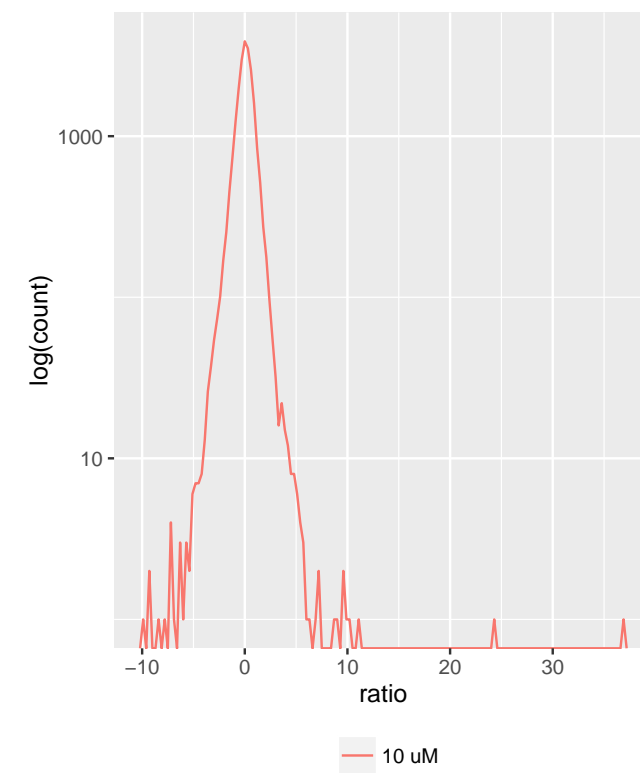

(e) MAPK dataset.

Supplement: S2 Fig — (a) The TCR dataset contains three proteomic experiments. The 5 min data describe the initiation of the TCR signaling pathway. The following response interfered lots of downstream proteins, resulting the 15 min data have the largest number of proteins among this dataset. Then the signal was transmitted to the nuclear and the amount of high-ratio proteins decreased, as described in the 60 min data. (b) The PKA dataset contains two proteomic experiments. The initiation of the cAMP signaling pathway came rapid, so the 1 min data almost illustrate all the following events. The 60 min data have fewer high-ratio proteins because the response of the signal had gradually vanished. (c) The myogenesis dataset contains two proteomic experiments. The 24 hr data have more differentially expressed proteins than the 72 hr data. (d) The CML dataset contains two proteomic data. Their distributions look quite alike, despite that the 50 nM treatment did down-regulate more proteins. Most proteins are down-regulated since the experiment aims to repress the BCR-ABL signaling pathway. (e) The MAPK dataset contains one gene expression experiment. The up-regulated probes slightly outnumber the down-regulated (54% versus 46%), and there are few probes of high ratios. (PDF) [file pcbi.1005601.s003.pdf]
