# Supplementary material for: A knowledge-based T2-statistic to perform pathway analysis for quantitative proteomic data
Source: PLoS Comput Biol. 2017 Jun 16;13(6):e1005601. doi: 10.1371/journal.pcbi.1005601 (PMC5493430; doi:10.1371/journal.pcbi.1005601)
Supplement: S1 Table — (PDF) [file pcbi.1005601.s004.pdf]

A knowledge-based T^2-statistic to perform pathway analysis for quantitative proteomic data

Supplementary Table S1

**Dataset**	**TCR**			**PKA**		**Myogenesis**		**CML**		**MAPK**
**Treatment**	**α-CD3ϵ**			**PGE2**		**Serum-free**		**Dasatinib**		**U0126**
	5 min	*15 min*	*60 min*	*1 min*	*60 min*	*24 hr*	*72 hr*	*5 nM*	*50 nM*	*10 μM*
**Number of Uniprot IDs**	30	376	330	594	595	2227	2227	5453	5443	21816
**STRING statistics**										
Mapped ENSP IDs	18	148	124	488	488	1920	1920	1404	1396	7542
PPI clusters – low	1	2	1	2	2	2	2	2	2	20
PPI clusters – medium	1	4	4	5	5	10	10	13	13	73
PPI clusters – high	1	6	7	17	17	73	73	38	38	167
PPI clusters – highest	3	10	7	14	14	74	74	40	40	192
**HitPredict statistics**										
Mapped Uniprot IDs	22	150	120	458	459	2019	2019	1546	1543	7913
PPI clusters – low	1	13	14	15	15	26	26	15	15	100
PPI clusters – high	1	13	14	17	17	28	28	20	20	116
**KEGG: 290 pathways**										
Mapped Uniprot IDs	22	122	104	328	328	1291	1291	758	756	4067
Mapped pathways	108	175	183	195	195	272	272	230	230	290
Enriched – $T^2 \times ST$	13	57	36	45	47	54	75	111	119	118
Enriched – $T^2 \times HP$	14	59	17	49	51	56	70	113	121	117
Enriched – DPA	66	68	71	15	19	73	68	15	12	17
Enriched – GSEA	69	60	43	8	6	31	39	29	33	4
Enriched – DAVID	47	53	35	28	28	73	73	54	54	119
**Reactome: 1618 pathways**										
Mapped Uniprot IDs	20	139	121	417	417	1601	1601	936	934	5426
Mapped pathways	191	420	404	529	529	1021	1021	801	801	1506
Enriched – $T^2 \times ST$	14	53	32	64	63	106	122	120	136	227
Enriched – $T^2 \times HP$	14	53	29	68	63	108	119	132	146	231
Enriched – DPA	23	34	41	35	50	123	112	113	142	80
Enriched – GSEA	22	23	26	103	91	89	121	48	51	0
Enriched – DAVID	81	78	51	125	125	238	238	196	196	82
**Ingenuity®: 658 pathways**										
Mapped Uniprot IDs	29	373	328	585	586	2216	2216	4815	4809	19709
Mapped pathways	268	317	305	276	276	460	460	309	309	449
Enriched – IPA®	225	232	185	79	79	227	227	129	129	91
