# Supplementary material for: A knowledge-based T2-statistic to perform pathway analysis for quantitative proteomic data
Source: PLoS Comput Biol. 2017 Jun 16;13(6):e1005601. doi: 10.1371/journal.pcbi.1005601 (PMC5493430; doi:10.1371/journal.pcbi.1005601)
Supplement: S2 Table — The enriched pathways are the pathways that fulfilled the significance requirements described in Table 1. We listed the number of clusters using different confidence levels. The number of PPI clusters is calculated for clusters with at least two proteins. Anti-CD3ϵ is specific to CD3ϵ; PGE2 targets four EP receptors but the downstream is almost under control of cAMP; serum starvation may result in cell cycle arrest and turn on muscle regulatory factors to promote myogenesis; dasatinib mainly targets BCR-ABL but has about 10 other targets of different kinase families; U0126 is a highly selective inhibitor of MEK1 and MEK2. (PDF) [file pcbi.1005601.s005.pdf]

A knowledge-based T^2-statistic to perform pathway analysis for quantitative proteomic data

Supplementary Table S2

Dataset	Experiment	Pathway Title	T^2×ST	T^2×HP	DPA	GSEA	DAVID
TCR	5 min	Generation of second messenger molecules	1/14	1/14	-	-	1/81
	15 min	Regulation of actin dynamics for phagocytic cup formation	6/53	7/53	2/34	5/23	63/78
		Phosphorylation of CD3 and TCR zeta chains	10/53	9/53	-	-	4/78
		RAF/MAP kinase cascade	13/53	12/53	21/34	22/23	-
		PI5P, PP2A and IER3 Regulate PI3K/AKT Signaling	14/53	13/53	24/34	7/23	-
		ERK/MAPK targets	16/53	15/53	-	-	28/78
		MAPK3 (ERK1) activation	21/53	21/53	-	9/23	47/78
		Downstream signal transduction	23/53	23/53	-	-	-
	60 min	Nuclear import of Rev protein	7/32	5/29	41/41	-	3/51
PKA	1 min	Processing of DNA double-strand break ends	3/64	3/68	-	-	43/125
		Nonhomologous End-Joining (NHEJ)	4/64	4/68	-	-	37/125
		Rho GTPase cycle	6/64	6/68	-	-	63/125
		Rap1 signaling	21/64	23/68	9/35	25/103	84/125
		G alpha (s) signaling events	62/64	66/68	33/35	-	-
	60 min	G alpha (12/13) signalling events	17/63	17/63	24/50	67/91	117/125
		HDR through MMEJ (alt-NHEJ)	50/63	50/63	29/50	86/91	-
		mTORC1-mediated signalling	40/63	40/63	49/50	-	-
Myogenesis	24 hr	Integrin cell surface interactions	8/106	8/108	7/123	-	125/238
		ECM proteoglycans	10/106	10/108	17/123	86/89	117/238
		Collagen degradation	12/106	12/108	2/123	2/89	216/238
		Assembly of collagen fibrils and other multimeric structures	16/106	16/108	5/123	44/89	133/238
		Endosomal/Vacuolar pathway	54/106	56/108	12/123	8/89	-
		G1/S-Specific Transcription	77/106	79/108	-	-	-
	72 hr	Collagen biosynthesis and modifying enzymes	5/122	5/119	103/112	-	104/238
		Smooth Muscle Contraction	9/122	9/119	-	-	88/238
		Degradation of the extracellular matrix	16/122	16/119	-	-	-
		Striated Muscle Contraction	19/122	19/119	1/112	7/121	144/238
		Activation of Matrix Metalloproteinases	89/122	86/119	-	-	-
		Creatine metabolism	101/122	98/119	2/112	58/121	-
		Lysosomal glycogen catabolism	120/122	117/119	80/112	-	-
CML	5 nM	MAPK6/MAPK4 signaling	14/120	16/132	-	9/48	54/196
		MAP2K and MAPK activation	30/120	37/132	-	-	64/196
		Regulation of actin dynamics for phagocytic cup formation	34/120	41/132	-	-	-
		RAF/MAP kinase cascade	38/120	46/132	-	-	-
		Negative regulation of MAPK pathway	39/120	47/132	-	-	80/196
		MAPK3 (ERK1) activation	98/120	110/132	-	-	-
		PI5P, PP2A and IER3 Regulate PI3K/AKT Signaling	72/120	84/132	-	-	-

continued on next page

Dataset	Experiment	Pathway Title	$T^2 \times$ST	$T^2 \times$HP	DPA	GSEA	DAVID
	50 nM	Signaling by SCF-KIT	42/136	46/146	-	-	101/196
		Regulation of KIT signaling	64/136	69/146	-	-	79/196
		ERK/MAPK targets	73/136	79/146	-	-	181/196
MAPK	10 μM	G alpha (q) signalling events	4/227	4/231	-	-	-
		G alpha (s) signalling events	5/227	5/231	-	-	-
		TP53 Regulates Metabolic Genes	16/227	14/231	-	-	-
		PIP3 activates AKT signaling	-	21/231	-	-	63/82
		NOTCH1 Intracellular Domain Regulates Transcription	-	36/231	-	-	13/82
		G alpha (z) signalling events	44/227	43/231	-	-	-
		MAP2K and MAPK activation	57/227	57/231	-	-	-
		Pre-NOTCH Transcription and Translation	-	53/231	9/80	-	15/82
		Constitutive Signaling by NOTCH1 PEST Domain Mutants	27/227	24/231	-	-	4/82
		Constitutive Signaling by NOTCH1 HD+PEST Domain Mutants	28/227	25/231	-	-	5/82
		Activated NOTCH1 Transmits Signal to the Nucleus	67/227	68/231	-	-	-
		NOTCH2 Activation and Transmission of Signal to the Nucleus	89/227	87/231	-	-	-
		Signaling by NOTCH3	121/227	119/231	-	-	-
		Signaling by NOTCH4	122/227	120/231	-	-	-
		MAPK3 (ERK1) activation	-	-	-	-	47/82
		Notch-HLH transcription pathway	-	-	11/80	-	55/82
